# Microbial Oxidation of the Fusidic Acid Side Chain by *Cunninghamella echinulata*

**DOI:** 10.3390/molecules23040970

**Published:** 2018-04-21

**Authors:** Abdel-Rahim S. Ibrahim, Khaled M. Elokely, Daneel Ferreira, Amany E. Ragab

**Affiliations:** 1 Department of Pharmacognosy, Faculty of Pharmacy, Tanta University, Tanta 31527, Egypt; arsib16@hotmail.com; 2 Department of Pharmaceutical Chemistry, Faculty of Pharmacy, Tanta University, Tanta 31527, Egypt; kelokely@pharm.tanta.edu.eg; 3 Institute for Computational Molecular Science and Department of Chemistry, Temple University, Philadelphia, PA 19122, USA; 4 Department of BioMolecular Sciences, Division of Pharmacognosy, School of Pharmacy, The University of Mississippi, University, MS 38677-1848, USA; dferreir@olemiss.edu

**Keywords:** fusidic acid, *Cunninghamella echinulata*, C-26-oxidation, C-27-oxidation

## Abstract

Biotransformation of fusidic acid (**1**) was accomplished using a battery of microorganisms including *Cunninghamella echinulata* NRRL 1382, which converted fusidic acid (**1**) into three new metabolites **2**–**4** and the known metabolite **5**. These metabolites were identified using 1D and 2D NMR and HRESI-FTMS data. Structural assignment of the compounds was supported via computation of ^1^H- and ^13^C-NMR chemical shifts. Compounds **2** and **3** were assigned as the 27-hydroxy and 26-hydroxy derivatives of fusidic acid, respectively. Subsequent oxidation of **3** afforded aldehyde **4** and the dicarboxylic acid **5**. Compounds **2**, **4** and **5** were screened for antimicrobial activity against different Gram positive and negative bacteria, *Mycobacterium smegmatis*, *M. intercellulare* and *Candida albicans*. The compounds showed lower activity compared to fusidic acid against the tested strains. Molecular docking studies were carried out to assist the structural assignments and predict the binding modes of the metabolites.

## 1. Introduction

Fusidic acid (**1**) is a natural antibacterial steroid-like compound without any steroidal activity [1,2]. It was first isolated and identified from the fungus *Fusidium coccineum* [1,2] and introduced into the market in the 1960s as the corresponding sodium salt for clinical use. It has activity against Gram positive bacteria, particularly methicillin resistant *Staphylococcus aureus* (MRSA), and modest activity against anaerobic Gram-negative bacteria [3,4]. Fusidic acid (**1**) acts through inhibition of protein synthesis by binding to the elongation factor EF-G [5]. The specific and narrow spectrum of activity of fusidic acid makes it an ideal target for investigating possible biotransformation pathways and the effects of the metabolites on the activity spectrum and/or efficacy. Here, we explored the metabolic fate of fusidic acid using several organisms among which the fungus *Cunninghamella echinulata* was the most proficient in the biotransformation of this antibiotic.

**Figure 1:**
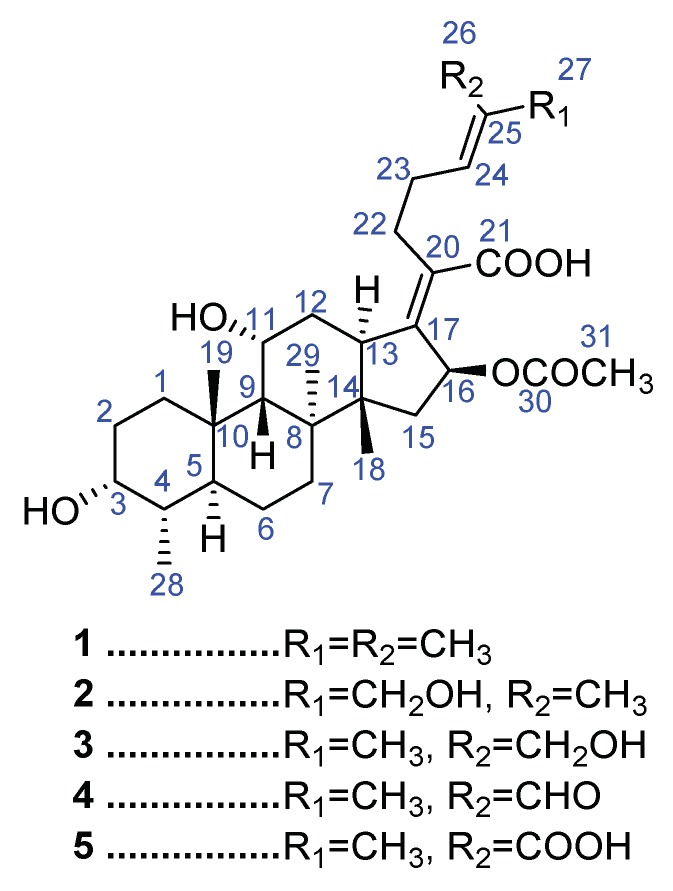
Structures of fusidic acid (**1**) and the isolated metabolites.

Fusidic acid (Figure 1) is metabolized into a dicarboxylic acid derivative in mammals. Other detected metabolites include 3-didehydrofusidic acid and fusidic acid 21-*O*-glucuronide conjugate [6,7]. Fusidic acid was reported to undergo oxygenation and oxidation by microbial transformation to yield 6-hydroxy, 7-hydroxy, 3-didehydro and 6-oxofusidic acid [8,9,10] or deacetylation to produce the 16β-hydroxy derivative, which spontaneously converts into the biologically inactive lactone analog [11]. 16-De-*O*-acetyl-7β-hydroxyfusidic acid was isolated from the fungus *Acremonium crotocinigenum* [12]. Biotransformation of the side chain functionalities of fusidic acid is rare. Several microbial strains were harnessed for studying the metabolism of drugs as a mimic of the phase-1 mammalian metabolism stage [13]. *Cunninghamella echinulata* is one of the established microbial models for investigating bioconversions of xenobiotics [13]. This study describes the formation of new metabolites emanating from microbial transformation of the side chain functionalities of fusidic acid using *C. echinulata*.

## 2. Results and Discussion

Compound **2** (Figure 1) showed a potassium adduct ion at *m*/*z* 571.3030 using high resolution electrospray ionization Fourier transform mass spectrometry (HRESI-FTMS) which, in conjunction with the ^1^H- and ^13^C-NMR data (Table 1 and Table 2), corresponds to a molecular formula of [C_31_H_48_O_7_ + K]^+^ (calculated 571.3032). The molecular formula of fusidic acid is C_31_H_48_O_6_ and the observed molecular formula of **2** thus indicates the mono-oxygenation of fusidic acid. By comparison of the ^13^C-NMR data of fusidic acid and compound **2**, C-27 was deshielded from δ_C_ 25.7 to 68.6 suggesting its conversion from CH_3_ to CH_2_-O-, and thus resulted in deshielding of C-25 and shielding of C-26 (Table 2).The DEPT 135 experiment showed nine methylene carbons compared to the eight of fusidic acid. The ^1^H-NMR spectrum of compound **2** in CDCl_3_ (Table 1) showed the presence of a singlet at δ_H_ 3.9 integrating for two protons with the absence of the singlet at δ_H_ 1.67 for Me-27 in the spectrum of fusidic acid. This shift is consistent with a methylene group carrying an electronegative atom, thus, indicating the structure of compound **2** as 27-hydroxyfusidic acid. Other proton and carbon signals were highly similar to those of fusidic acid (Table 1 and Table 2). The 2D HSQC NMR spectrum showed correlation of the proton singlet of CH_2_-27 (δ_H_ 3.9) and C-27 at δ_C_ 68.6 which confirmed the site of oxygenation in compound **2** at C-27 (Figure 1). The ^1^H-^1^H COSY spectrum of fusidic acid (Supplementary Materials) indicated the correlation of the protons at C-24 and C-27 which disappeared in the ^1^H-^1^H COSY spectrum of compound **2** suggesting that compound **2** is a (24*E*) isomer. The experimental and computed chemical shifts of compound **2** were compared to assign the degree of fitness (Supplementary Materials), using the mean absolute error (MAE) and regression analysis (R^2^) for that purpose. The absolute error for the computed ^13^C-NMR data of **2** was calculated as 68.14 and the MAE as 2.198 supporting the assignment of **2** as the (24*E*) geometrical isomer of the new 27-hydroxyfusidic acid.

The HRESI-FTMS data of compound **3** showed a potassium adduct ion at *m*/*z* 571.3030 which, in conjunction with ^13^C-NMR data, corresponds to a molecular formula of [C_31_H_48_O_7_ + K]^+^ (calculated 571.3032) suggesting the oxygenation of fusidic acid. Comparing the ^13^C-NMR data of fusidic acid and compound **3**, C-26 was deshielded from δ_C_ 17.8 to 61.2 which resulted in deshielding of C-24, C-25 and shielding of C-27 (Table 2). The DEPT 135 spectrum showed nine methylene carbons with the chemical shift of the carbon at δ_C_ 61.2 suggesting oxygenation at C-26. The ^1^H-NMR data of compound **3** in CDCl_3_ showed two doublets at δ_H_ 4.03 and 4.13 (^3^*J* = 11.75 Hz), characteristic for geminal coupling, replacing the singlet (δ_H_ 1.60) for Me-26 in the spectrum of fusidic acid. This shift is reminiscent of a methylene group attached to an electronegative atom suggesting the structure of compound **3** as 26-hydroxyfusidic acid. Other proton and carbon signals were similar to those of fusidic acid (Table 1 and Table 2). The gradient HMQC data showed correlation of the proton doublets at δ_H_ 4.03 and 4.13 and C-26 (δ_C_ 61.2) which confirmed the site of oxygenation in compound **3** at C-26 (Figure 1). The ^1^H-^1^H COSY spectrum showed the correlation of the protons at C-24 and C-27, indicating that compound **3** is a (24*Z*) isomer. The computed ^13^C-NMR spectrum of **3** showed an absolute error of 83.157 with an MAE of 2.682 matching the assignment of the structure of compound **3** as the new (24*Z*)-26-hydroxyfusidic acid.

**Figure 2:**
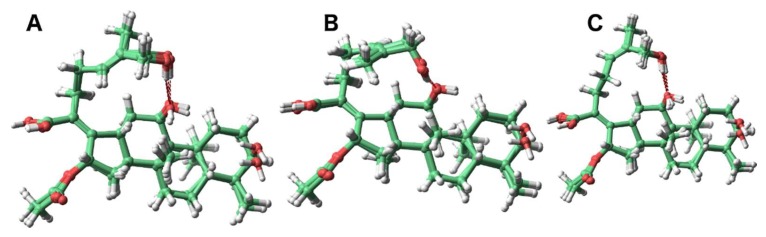
The most abundant conformers of **2** (**A** and **B**) and **3** (**C**).

We next investigated the phenomenon of the 27-hydroxymethylene protons in **2** resonating as a singlet and the 26-hydroxymethylene protons in **3** as two one-proton doublets. The lowest energy conformers were analyzed to investigate the relative chemical environment of these protons in each case (Figure 2). Owing to strong hydrogen bonding between the C-11 and C-27 hydroxy groups, two major orientations of the C-27 protons of compound **2** were observed (Figure 2, panels A and B). This creates similar average chemical environments and results in a singlet resonance for the geminal hydrogen atoms. In **3**, the hydrogen bonding between the C-11 and C-26 hydroxy groups anchored the C-26 methylene (Figure 2, panel C) group to such an extent as to create diastereotopic-like protons culminating in two one-proton doublets in the ^1^H-NMR spectrum. 

The HRESI-FTMS data of compound **4** revealed a sodium adduct ion at *m*/*z* 553.3131 which, in conjunction with the ^13^C-NMR data, accounts for a molecular formula of [C_31_H_46_O_7_ + Na]^+^ (calculated 553.3135), again indicative of the presence of an oxidation product of fusidic acid. By comparison of the ^13^C-NMR data of fusidic acid and compound **4**, C-26 was deshielded from δ_C_ 17.8 to 195.8 indicating the oxidation of Me-26 into a formyl group which resulted in deshielding of C-24, C-25 and shielding of C-27 (Table 2). The DEPT 90 spectrum of compound **4** showed nine methine carbons in contrast to the eight of fusidic acid. The ^1^H-NMR data of compound **4** in CDCl_3_ showed the presence of a one-proton singlet at δ_H_ 9.36 and the absence of the Me-26 singlet (δ_H_ 1.60) in the spectrum of fusidic acid. This shift is reminiscent of formyl group formation suggesting the structure of compound **4** as 26-formylfusidic acid. The HSQC spectrum showed correlation of the proton singlet (δ_H_ 9.36) and C-26 (δ_C_ 195.8), and, thus, confirmed the structure of the new compound **4** (Figure 1). The ^1^H-^1^H COSY spectrum showed the correlation of the protons at C-24 and C-27, indicating that compound **4** is a (24*Z*) isomer. The calculated absolute error, MAE and (R^2^) supported the structural assignment of the new compound **4** as (24*Z*)-26-formylfusidic acid (Supplementary Materials).

The molecular formula of compound **5** was determined as C_31_H_46_O_8_ via its ^13^C-NMR and HRESI-FTMS data which showed a sodium adduct ion at *m*/*z* 569.3072 for [C_31_H_46_O_8_ + Na]^+^ (calculated 569.3084). The molecular formula of compound **5** has one extra oxygen atom compared to compound **4** which is reminiscent of an oxidation product of fusidic acid. By comparison of the ^13^C-NMR data of fusidic acid and compound **5**, the C-26 resonance was deshielded from δ_C_ 17.8 to 172.9 which strongly suggests oxidation at C-26, thus resulted in deshielding of C-24, C-25 and shielding of C-27 (Table 2). The DEPT 90 and 135 spectra of compound **5** evidenced one fewer methyl group compared to fusidic acid which implied the presence of a hydroxycarbonyl functional group. The ^1^H-NMR data of compound **5** in methanol-*d*_4_ showed the disappearance of the Me-26 singlet (δ_H_ 1.60) present in the spectrum of fusidic acid. This is consistent with the presence of a carboxylic group, and hence the structure of compound **5** as 26-carboxyfusidic acid (Figure 1) which matched the literature data [7]. 2D NMR data of compound **5** supported the deduced structure.

Compounds **3** and **4** may be considered as intermediates towards the formation of compound **5** and this is the first report of their formation and structural elucidation. Von Daehne et al. reported as “unpublished observations” that compound **2** was chemically synthesized by Godtfredsen and Vangedal via oxidation of fusidic acid with selenium oxide in *t*-butanol [14], followed by reduction with sodium borohydride to yield compound **2**. The oxygenation step of the 26-Me and 27-Me diastereotopic ligands in the side chain of fusidic acid using *C. echinulata* does not exhibit regioselectivity, whereas subsequent oxidation of the mixture of **2** and **3** into the formyl and hydroxycarbonyl fusidic acid derivatives **4** and **5** proceeded regiospecifically at C-26.

The antimicrobial activity testing of compounds **2**, **4** and **5** revealed that oxidation of fusidic acid at C-26 to the formyl derivative **4** diminishes the activity, whilst further oxidation to the carboxylic acid **5** abolishes the activity completely. The oxygenation at C-27 decreased the antimicrobial activity of fusidic acid (Table 3). These results showed that the methyl groups in the side chain of fusidic acid are crucial for maximum activity.

**Figure 3:**
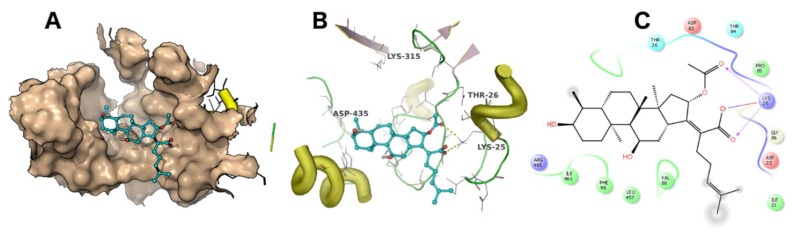
The binding mode of fusidic acid (**1**). The ligand binding pocket is shown as surface (**A**). The amino acid residues involved in ligand interaction are shown as lines (**B**). A 2D ligand interaction profile is demonstrated in (**C**).

**Figure 4:**
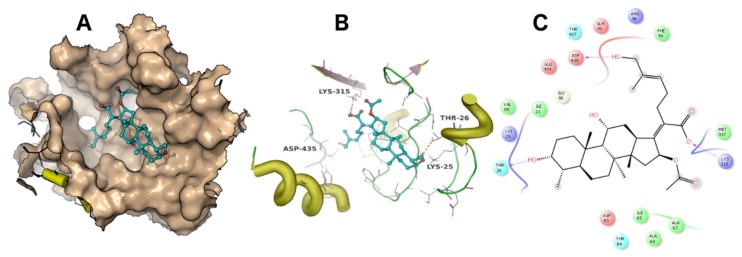
The binding mode of **2**. The ligand binding pocket is shown as surface (**A**). The amino acid residues involved in ligand interaction are shown as lines (**B**). A 2D ligand interaction profile is demonstrated in (**C**).

**Figure 5:**
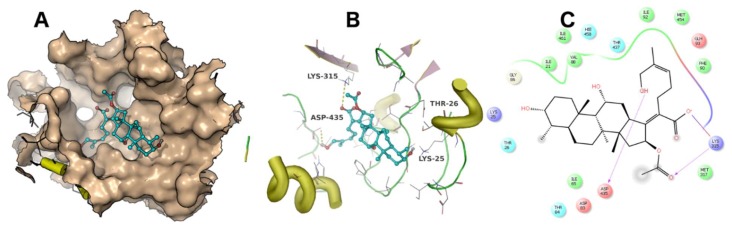
The binding mode of **3**. The ligand binding pocket is shown as surface (**A**). The amino acid residues involved in ligand interaction are shown as lines (**B**). A 2D ligand interaction profile is demonstrated in (**C**).

**Figure 6:**
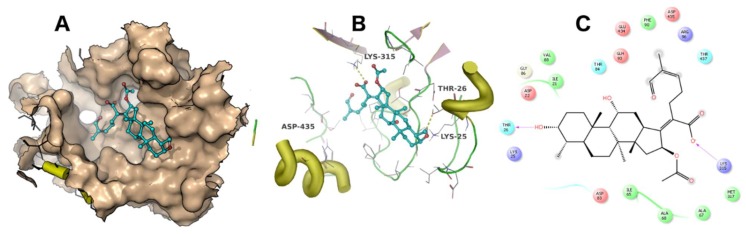
The binding mode of **4**. The ligand binding pocket is shown as surface (**A**). The amino acid residues involved in ligand interaction are shown as lines (**B**). A 2D ligand interaction profile is demonstrated in (**C**).

A docking simulation was carried out using the crystal structure of *Thermus thermophilus* EF-G (PDB accession code: 4V5F). Fusidic acid showed the best docking score of −4 kcal/mol, while compounds **2**, **3**, **4** and **5** exhibited docking scores of −2.5, −2.6, −2.8 and −0.36 kcal/mol, respectively. The simulated binding poses of compounds **2**, **3** and **4** were studied and compared with that of fusidic acid (Figure 3, Figure 4, Figure 5 and Figure 6). Compounds **1**, **2**, **3** and **4** exhibited non-covalent interactions with the amino acid residues of the ligand binding pocket, mostly in the form of electrostatic and Van der Waals contacts. 

The amino acid residues involved in ligand interaction include Thr26, Lys25, Ile21, Val88, Arg96, Asp435, Glu434, Met317, Lys315, Ala68, Ile65, Ala67, Asp83, Thr84, Thr437 and Phe90. Lys315 and Thr26 form conserved hydrogen bonds, while Asp435 forms a hydrogen bond only with **2**. Lys25 showed a strong ionic interaction with **1**. This simulation indicated that fusidic acid fits best in the binding pocket with non-covalent and ionic interactions, while compounds **2**–**4** showed less binding affinity which may account for their decreased activity. The docking score of compound **5** (−0.36 kcal/mol) implies weak or no binding which explains the complete loss of activity.

## 3. Materials and Methods 

### 3.1. General Experimental Procedures 

Sodium fusidate was purchased from Leo Pharmaceutical Company (Ballerup, Denmark). IR spectra were recorded on a Perkin Elmer IR spectrophotometer (PerkinElmer Inc., Waltham, MA, USA). UV data were acquired using a 60/PC ultraviolet spectrophotometer (Shimadzu, Kyoto, Japan). NMR spectra were recorded using Varian XL300 (Varian Inc., Palo Alto, CA, USA) and Bruker Avance 500 spectrophotometers (Bruker, Billerica, MA, USA) using CDCl_3_ and methanol-*d*_4_ as solvents and tetramethyl silane (TMS) as internal standard. ^1^H-NMR spectra were recorded at 300 or 500 MHz, and ^13^C-NMR spectra at 75 or 125 MHz. DEPT, COSY and HETCOR analyses were obtained using Varian Pulse Sequences at 300 or 500 MHz. HR-ESIFTMS data were acquired using a Bruker Bioapex FT-mass spectrometer (Bruker, Billerica, MA, USA) in ESI mode. Thin layer chromatography (TLC) was carried out using precoated silica gel 60 F_254_ plates (0.25 mm layer, E. Merck, Darmstadt, Germany) and visualization was by spraying with *p*-anisaldehyde reagent followed by heating at 110 °C.

### 3.2. Preparation of Fusidic Acid

Sodium fusidate was dissolved in water (50 mg/mL) and acidified with acetic acid. The precipitated fusidic acid was filtered, washed acid-free with distilled water, and dried to constant weight in a vacuum desiccator. The NMR and MS data were identical to reported data [14,15].

### 3.3. Microorganisms and Culture Conditions

Microbial transformation experiments were conducted according to published procedures [16]. For the initial screening experiments, 25 microbial cultures belonging to the genera *Aspergillus*, *Candida*, *Cunninghamella*, *Saccharomyces*, *Rhizopus*, *Penicillium*, *Streptomyces*, *Gymnascella*, *Lindera*, and *Rhodotorula* were used. The tested strains were obtained from either The American Type Culture Collection (ATCC, Manassas, VA, USA) or the National Center for Agricultural Utilization Research (NCAUR, Peoria, IL, USA). The strains were maintained at 4 °C on Sabouraud dextrose agar slants and subcultured quarterly.

### 3.4. Culture Media

In all fermentations, the medium consists of 10 mL/L glycerol, 10 g/L glucose, 5 g/L peptone, 5 g/L yeast extract, 5 g/L NaCl, and 5 g/L K_2_HPO_4_ in distilled water. The pH was adjusted to 6.0 before autoclaving at 121 °C for 15 min.

### 3.5. Initial Biotransformation Screening Experiments

Cells of the tested microorganisms were transformed from two-week old slants into sterile liquid medium (50 mL/250 mL flask) and kept on a gyratory shaker at 28 °C and 200 rpm for 72 h to give stage I culture. Stage I culture (5 mL) was used as an inoculum for stage II culture (50 mL/250 mL flask). After 24 h of incubation of stage II culture, sodium fusidate (10 mg) was added as a solution in absolute ethanol (250 μL) to each flask. Samples were taken after 3 and 6 days of incubation, acidified with a few drops of 10% HCl, filtered and the filtrate was extracted with an equal volume of chloroform. After evaporation of the chloroform, the residues were chromatographed on precoated silica gel plates using chloroform-methanol (5:1) or benzene-ethyl acetate- formic acid (3 mL:7 mL:1 drop) as mobile phase and detection was carried out by UV light visualization and *p*-anisaldehyde spray reagent. Both substrate and organism-free controls were also prepared and processed in the same way. The results of preliminary screening using fusidic acid were identical to those of using sodium fusidate. Amongst the tested strains, *C. echinulata* NRRL 1382 and *C. elegans* 1392 displayed the best transformations. This paper discusses the metabolites obtained from transformation using *C. echinulata*.

### 3.6. Large Scale Fermentation

Stage I cultures were prepared by inoculating culture media with two weeks old Sabouraud dextrose agar slants of *C. echinulata* and incubated at 28 °C, and 200 rpm for 72 h. Stage II cultures were initiated by inoculating stage I culture (5 mL) into new culture media (50 mL in 250 mL flasks) and incubated at 28 °C, and 200 rpm for 24 h. Sodium fusidate, dissolved in absolute ethanol (2.7 g/67 mL), was added to 270 stage II cultures to give a 0.02% *w*/*v* final concentration, and incubation continued for six days. Substrate and organism free control cultures were prepared. The cultures were pooled, acidified with 10% HCl (1 mL/30 mL culture), filtered and the filtrate was extracted twice with an equal volume of chloroform. The chloroform extract was dried over anhydrous sodium sulfate and evaporated under vacuum to give an amber-colored residue (3.4 g). TLC was carried out using chloroform-methanol (5:1) or benzene-ethyl acetate-formic acid (3 mL:7 mL:1 drop) as mobile phases and detection was carried out by UV light visualization and *p*-anisaldehyde spray reagent. 

### 3.7. Isolation of Metabolites

The residue obtained from the chloroform extract after evaporation (3.4 g) was loaded onto a silica gel column (300 g) and eluted with a gradient of ethyl acetate in benzene (0–60%) containing 0.2% formic acid and fractions of 100 mL were collected. The percentage of formic acid was increased to 0.4% starting from fraction no. 107 and similar fractions were pooled to give three groups of fractions.

#### 3.7.1. Fractions 80–106

The residue obtained upon pooling and evaporation of these fractions (360 mg) was rechromatographed on a silica gel column (40 g) using a gradient of methanol/chloroform (0–10%), and 50 mL fractions were collected. Fractions 47–64 afforded compound **4** (110 mg) and fractions 73–136 gave compound **5** (72 mg).

#### 3.7.2. Fractions 122–142

The residue of these fractions (300 mg) was rechromatographed on a silica gel column (40 g) using a gradient of methanol/chloroform (0–10%) and 50 mL fractions were collected. Fractions 46–84 afforded compound **2** (115 mg).

#### 3.7.3. Fractions 143–190

The residue of these fractions (320 mg) was partially purified using Sephadex LH-20 column (200 mL bed volume) chromatography followed by silica gel column chromatography (40 g) using a gradient of methanol/chloroform (0–4%) and collecting 50 mL fractions. Fractions 79–114 yielded compound **3** which was recrystallized from n-hexane/chloroform mixture to provide 21 mg of pure **3**.

#### 3.7.4. 27-Hydroxyfusidic Acid (**2**)

White powder; UV (MeOH) λ_max_ 223 nm; IR ν_max_ (KBr disc) cm^−1^: 3440, 2880, 1725, 1395, 1275; ^1^H and ^13^C-NMR (CDCl_3_): see Table 1 and Table 2; HRESI-FTMS (*m*/*z*): 571.3030 [M + K]^+^ (calc. for C_31_H_48_O_7_K, 571.3032).

#### 3.7.5. 26-Hydroxyfusidic Acid (**3**)

White powder; UV (MeOH) λ_max_ 223 nm; IR ν_max_ (KBr disc) cm^−1^: 3432, 2936, 1717, 1638, 1443, 1379, 1260; ^1^H and ^13^C-NMR (CDCl_3_): see Table 1 and Table 2; HRESI-FTMS (*m/z*): 571.3030 [M + K]^+^ (calc. for C_31_H_48_O_7_K, 571.3032).

#### 3.7.6. 26-Formylfusidic Acid (**4**)

White powder; UV (MeOH) λ_max_ 218 nm; IR *ν*_max_ (KBr disc) cm^−1^: 3500, 2970, 2910, 1720, 1690, 1465, 1385, 1265; ^1^H and ^13^C-NMR (CDCl_3_): see Table 1 and Table 2; HRESI-FTMS (*m*/*z*): 553.3131 [M + Na]^+^ (calc. for C_31_H_46_O_7_Na, 553.3135).

#### 3.7.7. 26-Carboxyfusidic Acid (**5**)

White powder; UV (MeOH) λ_max_ 223 nm; IR *ν*_max_ (KBr disc) cm^−1^: 3435, 3169, 2939, 1700, 1641, 1381, 1260; ^1^H and ^13^C-NMR (CDCl_3_): see Table 1 and Table 2; HRESI-FTMS (*m*/*z*): 569.3072 [M + Na]^+^ (calc. for C_31_H_46_O_8_Na, 569.3084).

### 3.8. Antimicrobial Activity

Samples were tested according to the National Committee of Clinical Laboratory Standard (NCCLS, 1994) using ATCC strains.

### 3.9. Assignment of Relative Configuration

To assign the relative configuration of the compounds, all possible chemical structures were sketched and energy minimized in Maestro. MacroModel with the OPLS3 forcefied was used to generate the conformers of the proposed structures. We used the stochastic conformational search approach of MacroModel and the Monte Carlo multiple minimum method to allow for better torsional sampling. The energy window for selecting the conformers was defined at 10.04 kcal mol^−1^. Geometry optimization and frequencies were calculated for all optimized conformers, based on Boltzmann analysis, using Gaussian 09 at the M06-2X/6-31+G(d,p) level. Gaussian 09 at the B3LYP/6-311+G(2d,p) level was used to compute the NMR shielding tensors using the gauge-independent (or including) atomic orbitals (GIAO) method. In all DFT calculations we used the integrated equation formalism polarized continuum model (IEFPCM) was used.

### 3.10. Protein Preparation

The protein crystal structure of *T. thermophilus* EF-G (PDB accession code: 4V5F) was obtained from the protein databank (www.rcsb.org). The protein structure was prepared for docking by PrepWizard of the Schrödinger suite. Missing hydrogen atoms, amino acid side chains and loops were added. To account for correction of hydrogen bond networks, the orientations of amide groups (Asn and Gln), hydroxy groups (Tyr, Thr and Ser), and protonation states of imidazole moiety (His) were adjusted. No energy minimization was conducted.

### 3.11. Ligand Preparation

The compounds were sketched and converted into 3D structures in Maestro. The molecules were then prepared to address all possible protonation and tautomerization states using LigPrep with the OPLS3 forcefield. Only the lowest energy conformer for each ligand was kept.

### 3.12. Receptor Grid Preparation

The make receptor module of OpenEye scientific software (www.eyesopen.com) was used to construct the receptor grid. The native ligand was used to define the centroid of the docking box. The volume and dimensions of the grid box were defined as 7374 Å^3^ (17.27 Å × 19.14 Å × 22.31 Å). The dimensions of the outer contour of the docking region was 3140 Å^3^.

### 3.13. Docking Simulation

The multi-conformers’ compound database was docked using FRED of the OpenEye scientific software with standard docking precision was used. One best pose was saved for each compound. 

## 4. Conclusions

Among the screened strains, *C. echinulata* was the only organism that metabolized fusidic acid (**1**) in a regioselective fashion targeting the allylic Me-26 and the Me-27 groups of the hydrophobic side chain. The microorganism seems to detoxify the antibiotic fusidic acid (**1**) by regioselective oxidation of the methyl groups of the hydrophobic side chain into hydroxymethyl, formyl and hydroxycarbonyl functionalities in order to minimize the antimicrobial activity. The dicarboxylic acid may eventually undergo decarboxylation to norfusidic acid, which, however is yet to be isolated and assessed for antimicrobial activity. The intermediate oxidation products **2**–**4** may be exploited to develop antibiotic ligands with better activity and lower toxicity. These data indicate the presence of an interesting oxidation system in *C. echinulata* which targeted the side chain of fusidic acid in contrast to *C. elegans* which targeted ring B in our previous work [17].

## Figures and Tables

**Table 1:** ^1^H-NMR data of fusidic acid and the isolated metabolites. δ_H_ ppm (*J* = Hz).

Position	Compound				
	1 *	2 (300 MHz)	3 (500 MHz)	4 (300 MHz)	5 (500 MHz)
1	1.51(m)/2.17 (m)	1.49 (m)/2.07 (m)	1.50 (m)/2.09 (m)	1.50 (m)/2.09 (m)	1.51 (m)/2.17 (m)
2	1.75 (m)/1.86 (m)	1.71 (m)/1.82 (m)	1.63 (m)	1.70 (m)/1.83 (m)	1.62 (m)/1.88 (m)
3	3.76 (s)	3.72 (s)	3.73 (s)	3.75 (d, 1.54)	3.68 (s)
4	1.58 (m)	1.46 (brs)	1.51 (m)	1.46 (m)	1.55 (m)
5	2.11 (m)	2.17 (m)	2.17 (m)	2.10 (m)	2.17 (m)
6	1.13 (m)/1.59 (m)	1.09 (m)/1.60 (m)	1.12 (m)/1.60 (m)	1.11 (m)/1.70 (m)	1.16 (m)/1.73 (m)
7	1.12 (m)/1.74 (m)	1.68 (m)/1.11(m)	1.68 (m)/1.12(m)	1.24 (m)/1.83 (m)	1.16 (m)/1.8 (m)
8	-	-	-	-	-
9	1.57 (s)	1.55 (s)	1.59 (s)	1.56 (s)	1.62 (s)
10	-	-	-	-	-
11	4.35 (brs)	4.36(brs)	4.36 (brs)	4.34 (brs)	4.34 (brs)
12	1.85 (m)/2.33 (m)	1.82 (m)/2.44 (m)	2.44 (m)	1.87 (m)/2.43 (m)	1.89 (m)/2.32 (m)
13	3.06 (d, 10.91)	2.95 (d, 13.0)	3.06 (d, 10.66)	3.08 (d, 11.10)	3.11 (d, 11.20)
14	-	-	-	-	-
15	1.30 (d, 14.20)/2.19 (m)	1.27 (d, 14.0)/2.17 (m)	1.27 (m)/2.19 (m)	2.10 (m)/1.40 (m)	1.27(d, 4.20)/2.18 (m)
16	5.88(d, 8.32)	5.88 (d, 8.2)	5.86 (d, 7.02)	5.90 (d, 8.3)	5.85 (d, 8.17)
17	-	-	-	-	-
18	0.89 (s)	0.88 (s)	0.91 (s)	0.91 (s)	0.96 (s)
19	0.96 (s)	0.96 (s)	0.98 (s)	0.96 (s)	1.02 (s)
20	-	-	-	-	-
21	-	-	-	-	-
22	2.46 (m)	2.55 (m)	2.55 (m)	2.43 (m)/2.61 (m)	2.57(m)/2.65 (m)
23	2.07 (m)/2.17(m)	2.05 (m)/2.20 (m)	2.30 (m)	2.61 (m)	2.33 (m)
24	5.10 (t, 6.97)	4.49 (t, 7.2)	5.26 (t, 7.2)	6.49 (t, 7.9)	6.80 (t, 8.0)
25	-	-	-	-	-
26	1.60 (s)	1.62 (s)	4.03 (d, 11.75), 4.13 (d, 11.75)	9.36 (s)	-
27	1.67 (s)	3.9 (s)	1.77 (s)	1.73 (s)	1.84 (s)
28	0.90 (d, 5.8)	0.89 (d, 7.8)	0.90 (d,7.28)	0.90 (d, 6.31)	0.92 (d, 6.43)
29	1.38 (s)	1.34 (s)	1.38 (s)	1.36 (s)	1.41 (s)
31	1.96 (s)	1.97 (s)	1.99 (s)	1.96 (s)	1.98 (s)

* Data of fusidic acid (**1**) taken from reference [14].

**Table 2:** ^13^C-NMR data of fusidic acid and the isolated metabolites. δ_C_ ppm.

Position	Compound				
	1 *	2 (75 MHz)	3 (125 MHz)	4 (75 MHz)	5 (125 MHz)
1	30.2	30.1	30.2	30.5	31.4
2	29.8	30.4	30.1	30.2	31.4
3	71.5	71.9	72.0	71.9	72.9
4	36.4	36.1	35.7	36.7	38.6
5	36.0	37.1	37.2	36.3	37.2
6	20.9	21.4	23.0	21.2	22.8
7	32.1	32.1	32.0	31.9	33.3
8	39.5	49.1	39.9	39.3	41.1
9	49.3	50.0	49.8	49.7	51.1
10	36.9	39.8	36.9	37.3	38.2
11	68.2	69.0	68.5	68.5	69.0
12	35.6	35.5	35.7	36.0	37.8
13	44.3	44.7	44.5	44.9	45.6
14	48.7	49.1	49.2	49.2	50.4
15	38.9	39.2	39.3	39.3	40.4
16	74.5	74.9	74.9	74.8	76.1
17	150.7	150.2	150.4	152.8	150.6
18	17.8	18.0	18.0	18.3	18.5
19	23.0	23.7	23.6	23.4	24.2
20	129.6	129.9	130.2	128.8	131.6
21	174.4	173.9	173.2	173.2	174.0
22	28.8	27.2	28.1	29.6	29.0
23	28.5	27.9	28.8	27.7	30.3
24	123.1	124.0	127.1	152.2	142.5
25	132.6	136.2	135.6	140.3	130.2
26	17.8	14.1	61.2	195.8	172.9
27	25.7	68.6	21.8	9.6	13.0
28	15.9	16.3	16.3	16.3	16.9
29	23.9	24.1	24.0	24.3	24.0
30	170.7	171.4	171.6	171.2	172.0
31	20.6	21.0	21.0	21.0	21.1

* Data of fusidic acid (**1**) taken from reference [14].

**Table 3:** Antimicrobial activity testing of fusidic acid and the isolated metabolites.

Microorganism	Compound, MIC (μg/mL)			
	Fusidic acid 1	2	4	5
*Streptomyces faecalis*	1.50	50	50	-ve *
*Streptomyces durans*	6.00	25	25	-ve
*Staphyllococcus aureus*	0.38	2.5	2.5	-ve
*Bacillus subtlis*	0.38	100	50	-ve
*Escherichia coli*	-ve	-ve	-ve	-ve
*Pseudomonas aeruginosa*	-ve	-ve	-ve	-ve
*Mycobacterium smegmatis*	12.5	100	-ve	-ve
*Mycobacterium intercellulare*	12	-ve	-ve	-ve
*Candida albicans*	1.25	-ve	-ve	-ve

* -ve (no antimicrobial activity) at the highest tested concentration (100 μg/mL).

## Supplementary Materials

The following are available online. Figures S1–S26 are NMR and mass spectra of the isolated compounds. Excel sheets contain the NMR (proton and carbon) calculation results.
