# Supplementary material for: Microbial Oxidation of the Fusidic Acid Side Chain by Cunninghamella echinulata
Source: Molecules. 2018 Apr 21;23(4):970. doi: 10.3390/molecules23040970 (PMC6017311; doi:10.3390/molecules23040970)
Supplement: Supplementary file 1 [file molecules-23-00970-s001.zip › Supplementary/Revised supporting information .pdf]

Supplementary Data

Microbial oxidation of the fusidic acid side chain by *Cunninghamella echinulata*

Abdel-Rahim S. Ibrahim [1], Khaled M. Elokely [2,3], Daneel Ferreira [4] and Amany E. Ragab [1,*]

[1] Department of Pharmacognosy, Faculty of Pharmacy, Tanta University, Tanta; 31527, Egypt; arsib16@hotmail.com
[2] Department of Pharmaceutical Chemistry, Faculty of Pharmacy, Tanta University, Tanta; 31527, Egypt; kelokely@pharm.tanta.edu.eg
[3] Institute for Computational Molecular Science and Department of Chemistry, Temple University, Philadelphia; PA 19122, United States; kelokely@temple.edu
[4] Department of BioMolecular Sciences, Division of Pharmacognosy, School of Pharmacy, The University of Mississippi, University, MS 38677-1848, United States; dferreir@olemiss.edu
* Correspondence: amany.ragab@pharm.tanta.edu.eg ; Tel.: +20-40-333-6007-EXT 266

Contents	Page Number
^{1}H and ^{13}C NMR spectra of (24*E*)- 27-hydroxyfusidic acid (**2**)	S3
DEPT 135 spectrum of (24*E*)- 27-hydroxyfusidic acid (**2**)	S4
^{1}H-^{1}H COSY spectrum of fusidic acid (**1**)	S5
^{1}H-^{1}H COSY spectrum of (24*E*)- 27-hydroxyfusidic acid (**2**)	S6
HMBC spectrum of (24*E*)- 27-hydroxyfusidic acid (**2**)	S7
HRESI-MS spectrum of (24*E*)- 27-hydroxyfusidic acid (**2**)	S8
^{1}H and ^{13}C NMR spectra of (24*Z*)- 26-hydroxyfusidic acid (**3**)	S9
DEPT 135 spectrum of (24*Z*)- 26-hydroxyfusidic acid (**3**)	S10
^{1}H-^{1}H COSY spectrum of (24*Z*)- 26-hydroxyfusidic acid (**3**)	S11
HMQC spectrum of (24*Z*)- 26-hydroxyfusidic acid (**3**)	S12
HRESI-MS spectrum of (24*Z*)- 26-hydroxyfusidic acid (**3**)	S13
^{1}H and ^{13}C NMR spectra of 26-formylfusidic acid (**4**)	S14
DEPT 90 spectrum of 26-formylfusidic acid (**4**)	S15
^{1}H-^{1}H COSY spectrum of 26-formylfusidic acid (**4**)	S16
HMBC spectrum of 26-formylfusidic acid (**4**)	S17
HRESI-MS spectrum of 26-formylfusidic acid (**4**)	S18
^{1}H and ^{13}C NMR spectra of 26-carboxyfusidic acid (**5**)	S19
DEPT 90 and 135 spectra of 26-carboxyfusidic acid (**5**)	S20
^{1}H-^{1}H COSY spectrum of 26-carboxyfusidic acid (**5**)	S21
HMBC spectrum of 26-carboxyfusidic acid (**5**)	S22
HRESI-MS spectrum of 26-carboxyfusidic acid (**5**)	S23
A list of screened strains	S24

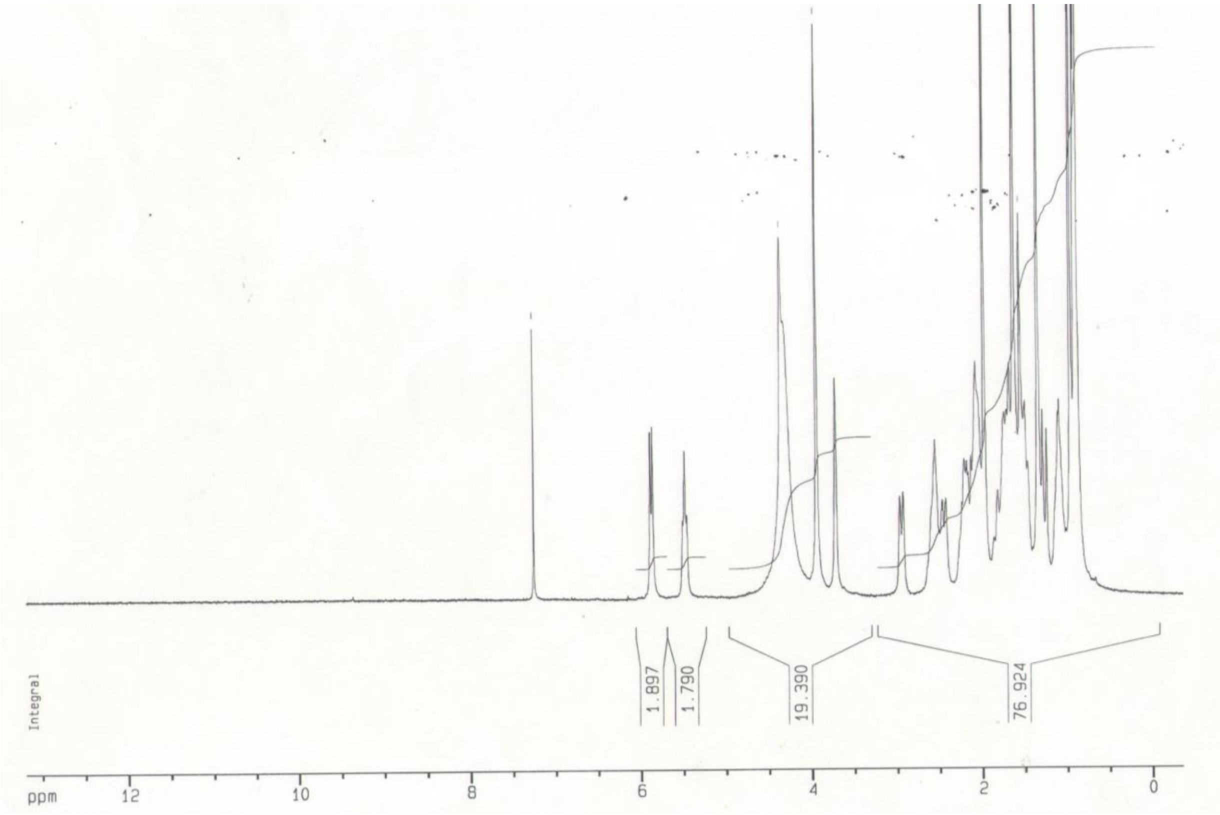

Figure S1. ^{1}H NMR spectrum of (24*E*)- 27-hydroxyfusidic acid (**2**), ($CDCl_3$, 300 MHz)

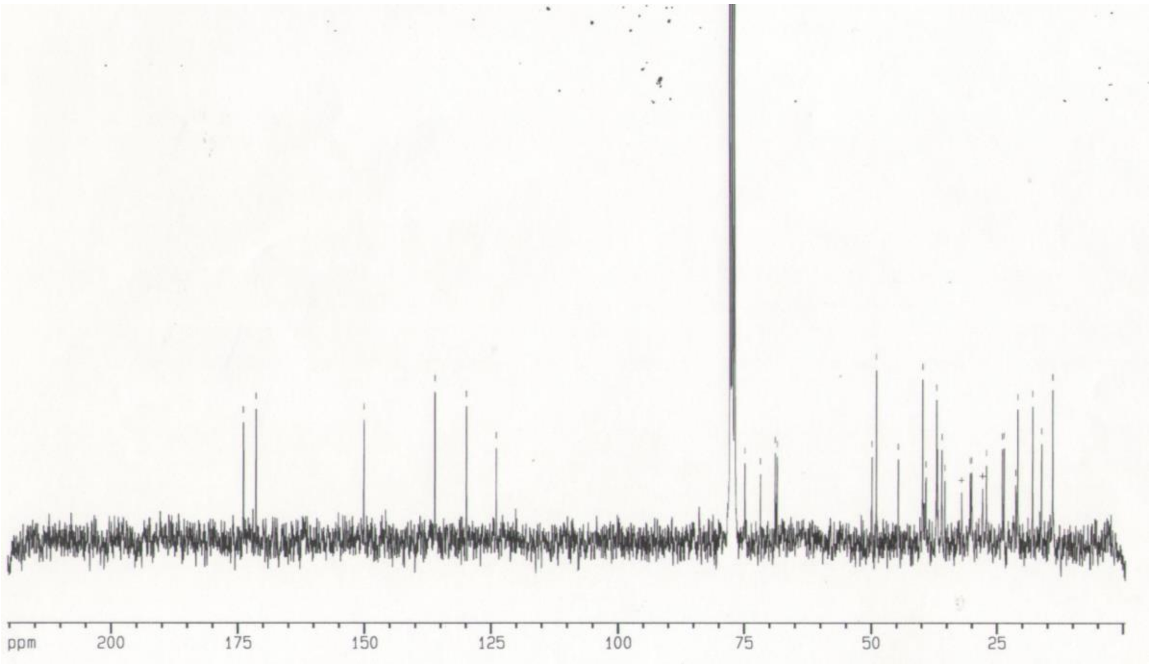

Figure S2. ^{13}C NMR spectrum of (24*E*)- 27-hydroxyfusidic acid (**2**), ($CDCl_3$, 75 MHz)

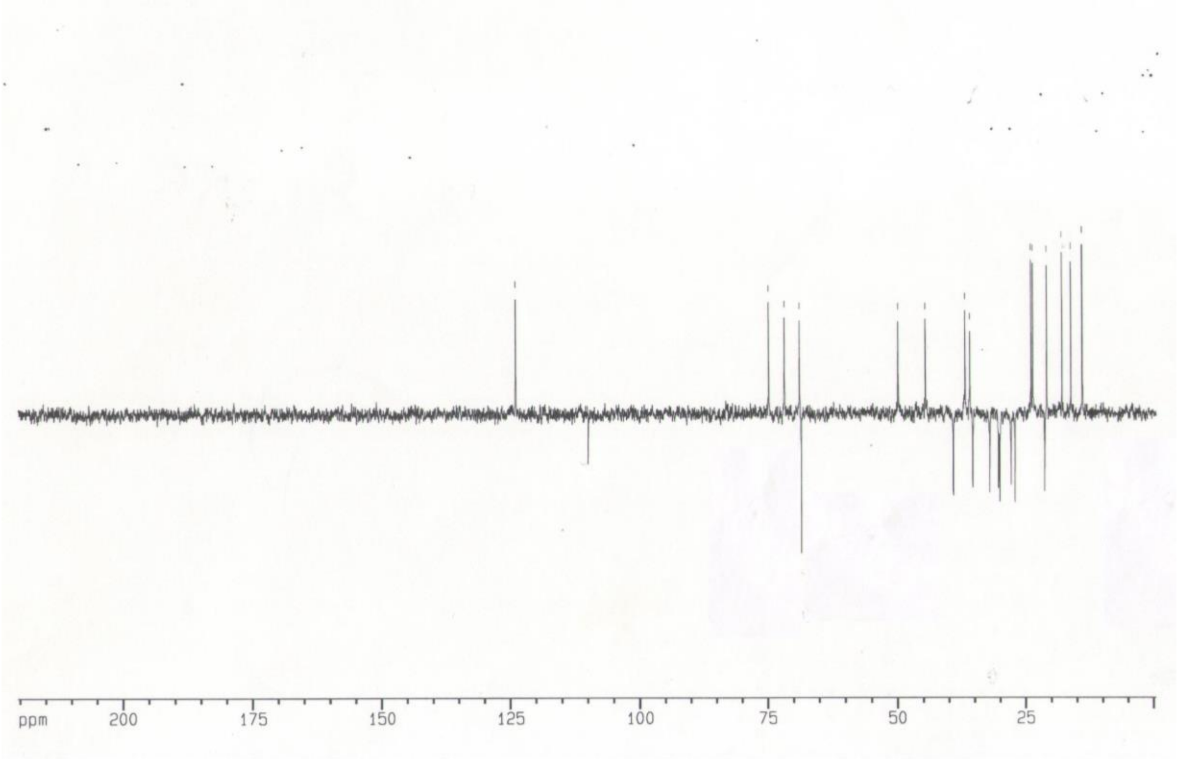

Figure S3. DEPT 135 spectrum of (24*E*)- 27-hydroxyfusidic acid (**2**)

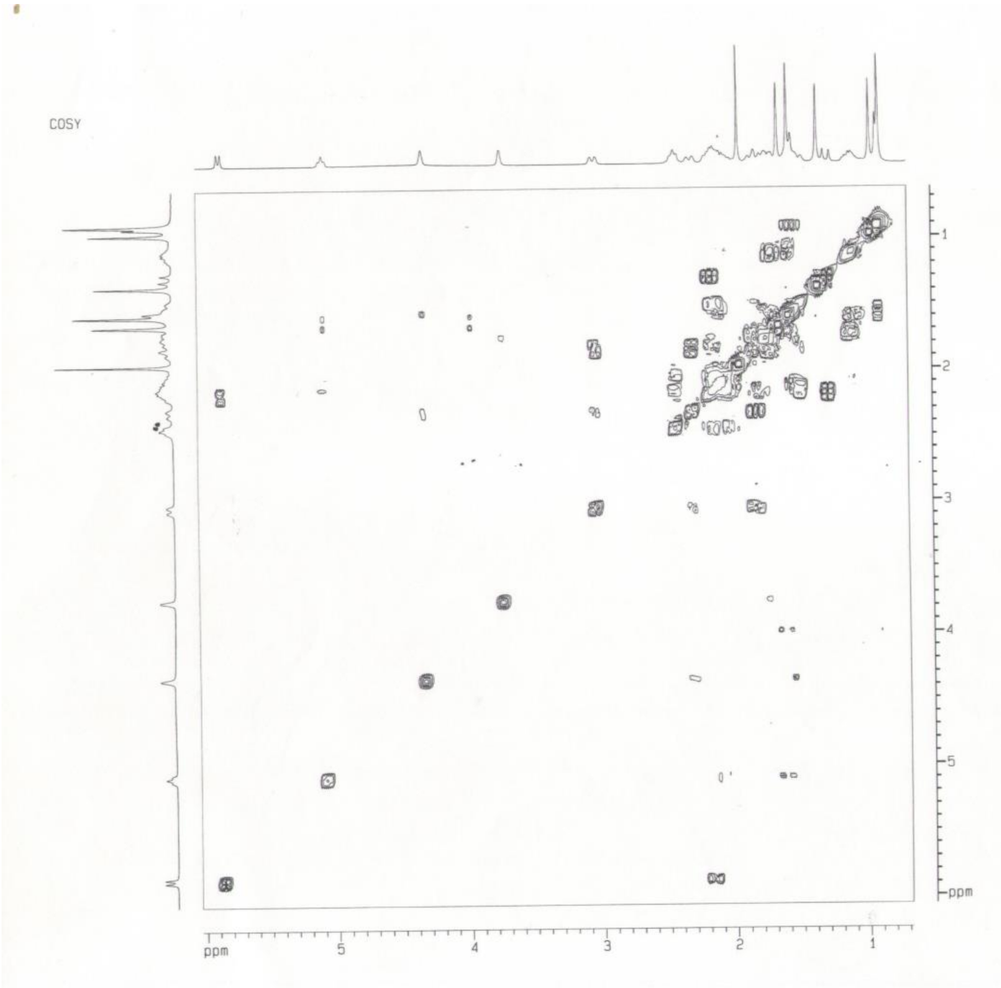

Figure S4. ^{1}H-^{1}H COSY spectrum of fusidic acid (**1**).

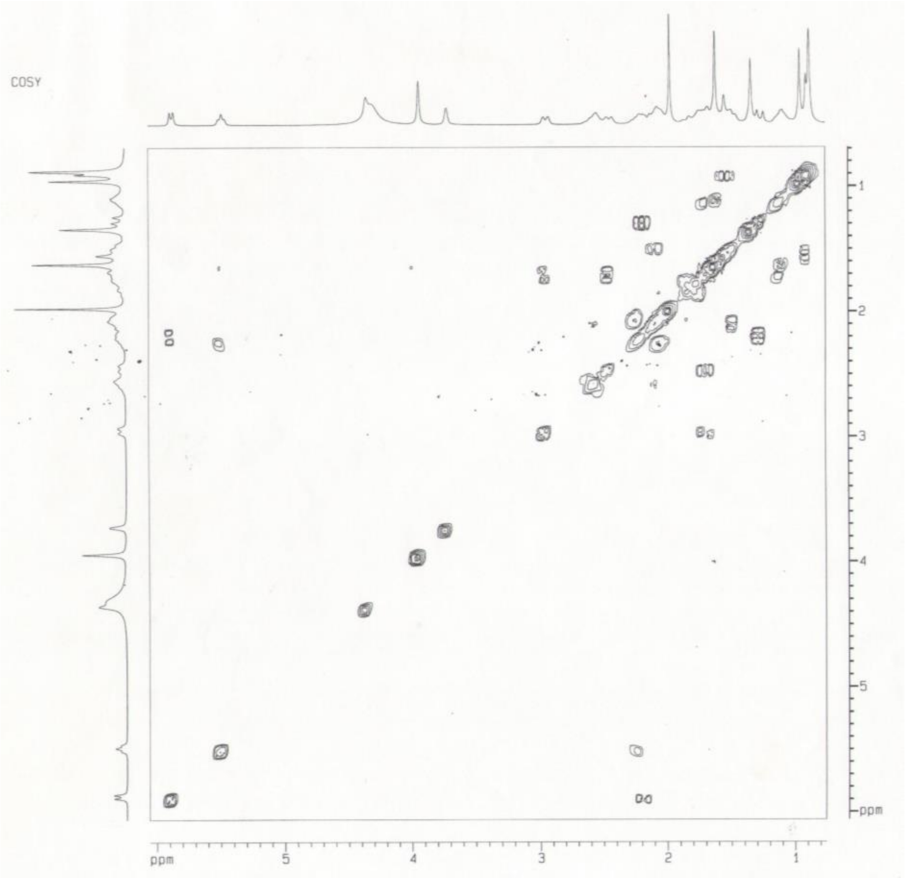

Figure S5. ^{1}H-^{1}H COSY spectrum of **(24*E*)- 27-hydroxyfusidic acid (**2**).**

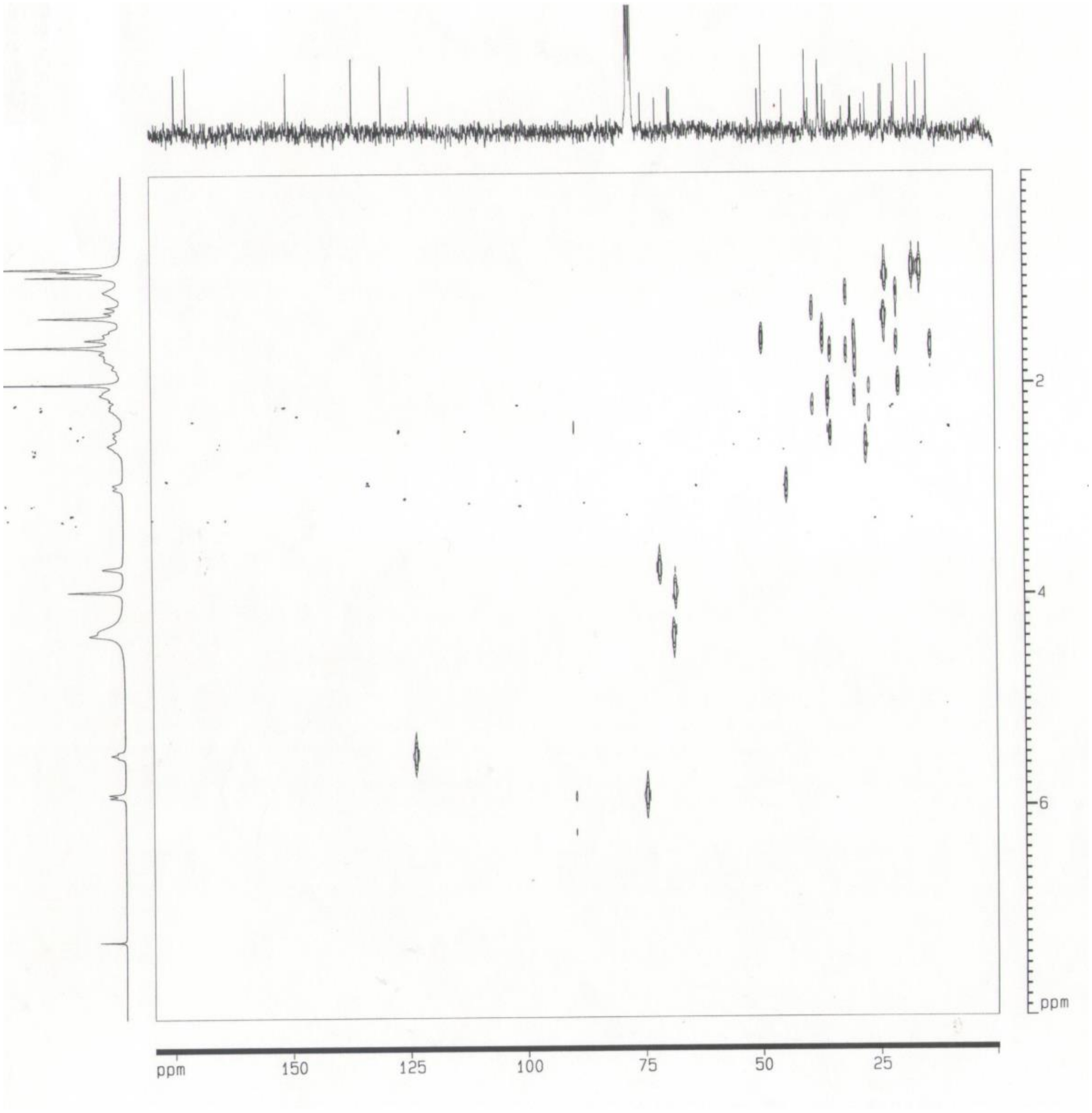

Figure S6. HSQC spectrum of (24*E*)- 27-hydroxyfusidic acid (**2**)

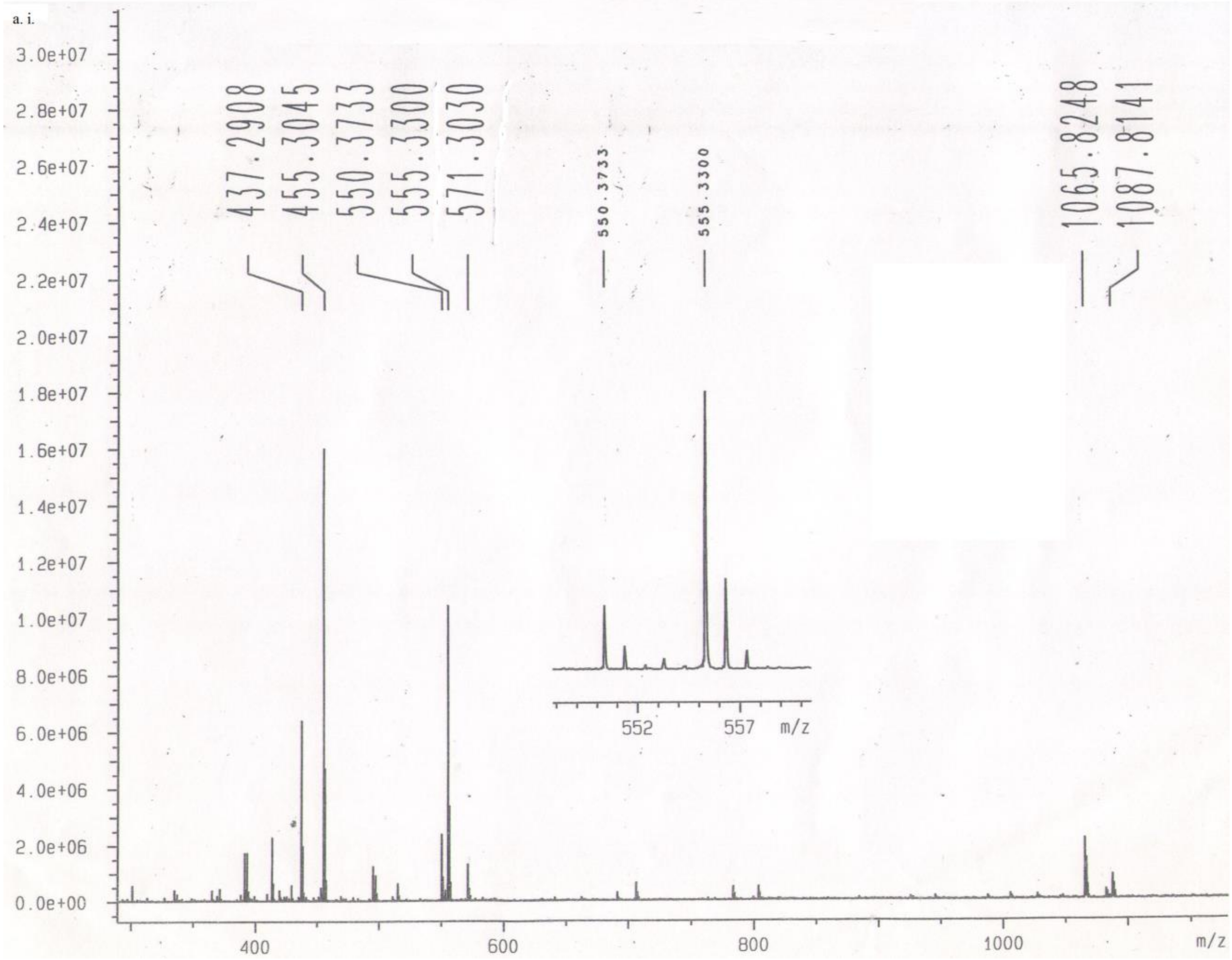

Figure S7. HRESI-MS spectrum of (24*E*)- 27-hydroxyfusidic acid (**2**)

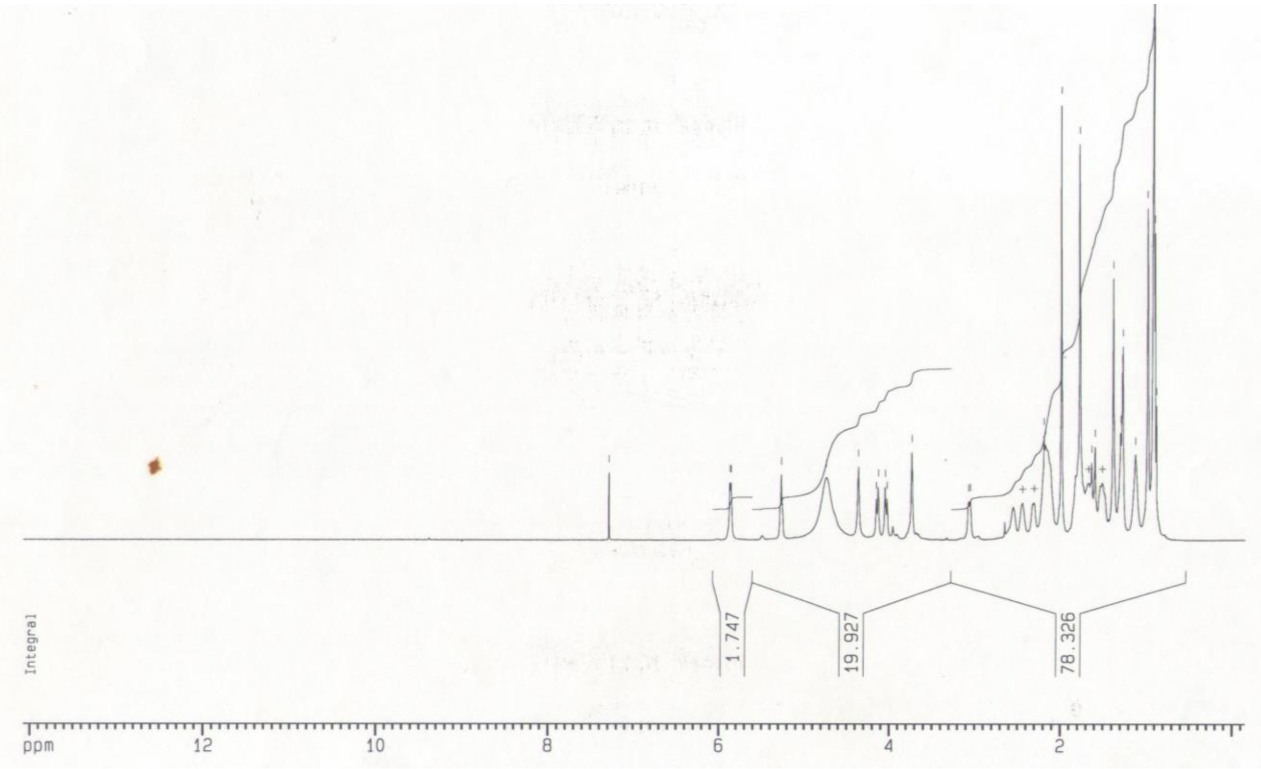

Figure S8. ^{1}H NMR spectrum of (24*Z*)- 26-hydroxyfusidic acid (**3**), ($CDCl_3$, 500 MHz)

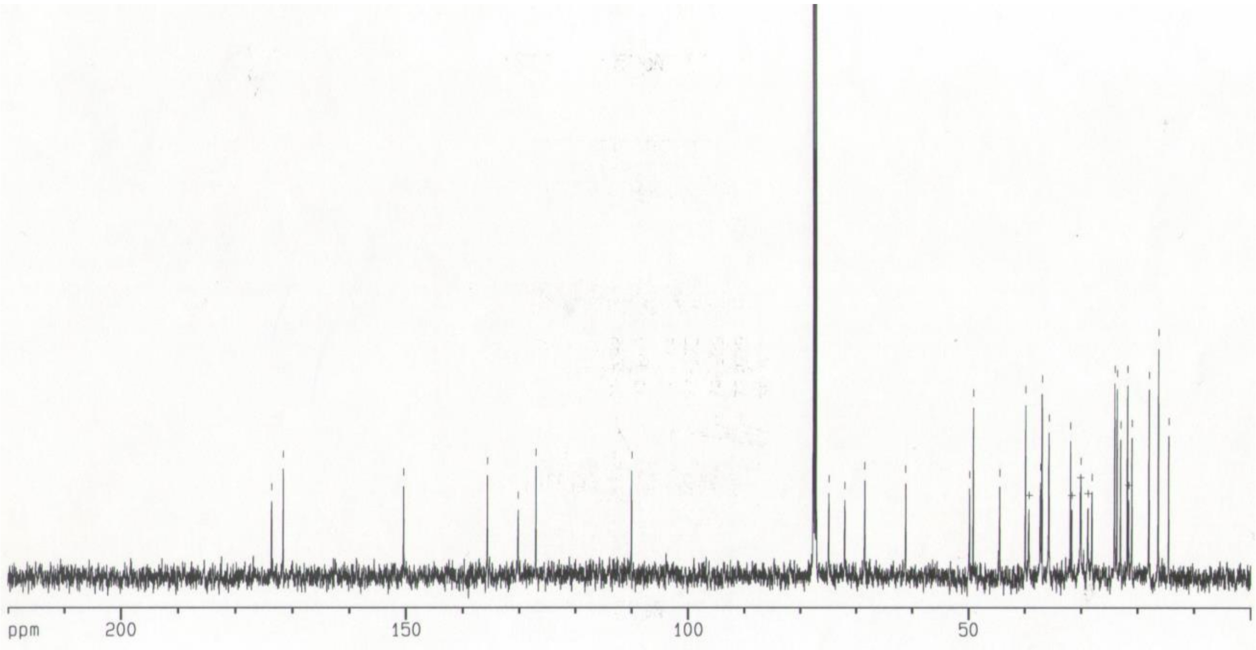

Figure S9. ^{13}C NMR spectrum of (24*Z*)- 26-hydroxyfusidic acid (**3**), ($CDCl_3$, 125 MHz)

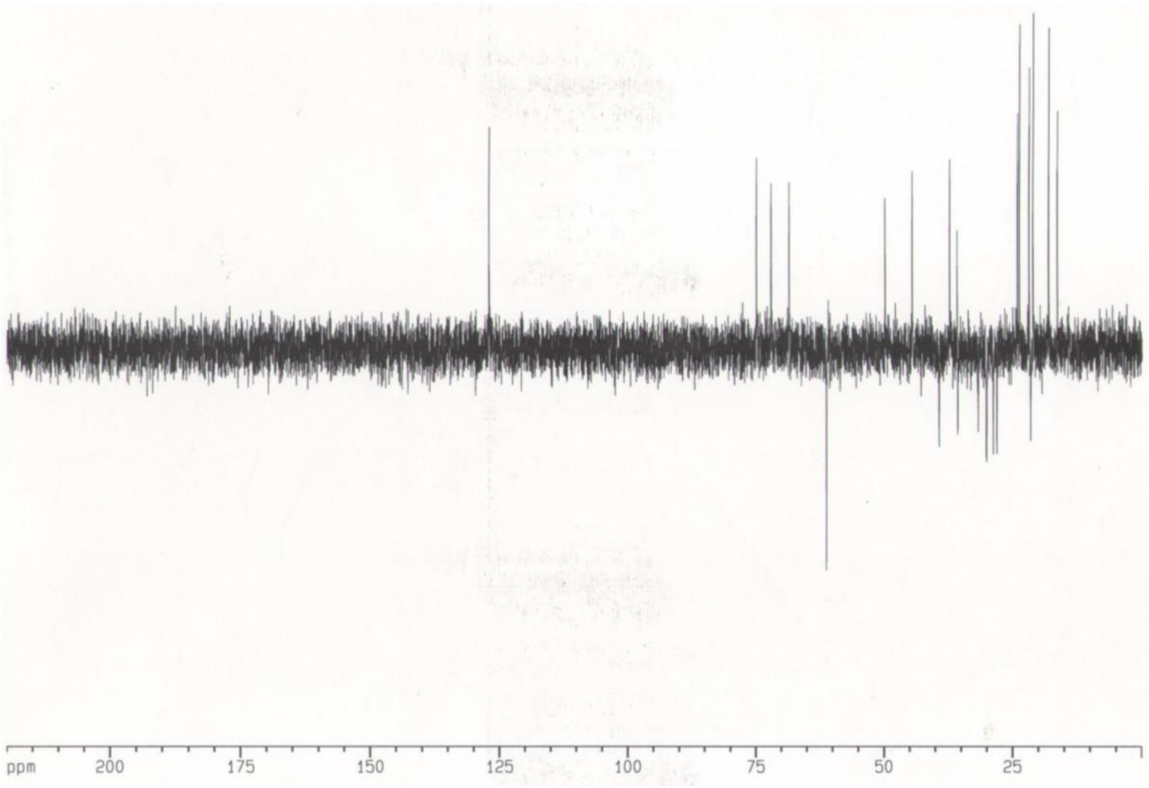

Figure S10. DEPT 135 spectrum of (24*Z*)- 26-hydroxyfusidic acid (**3**).

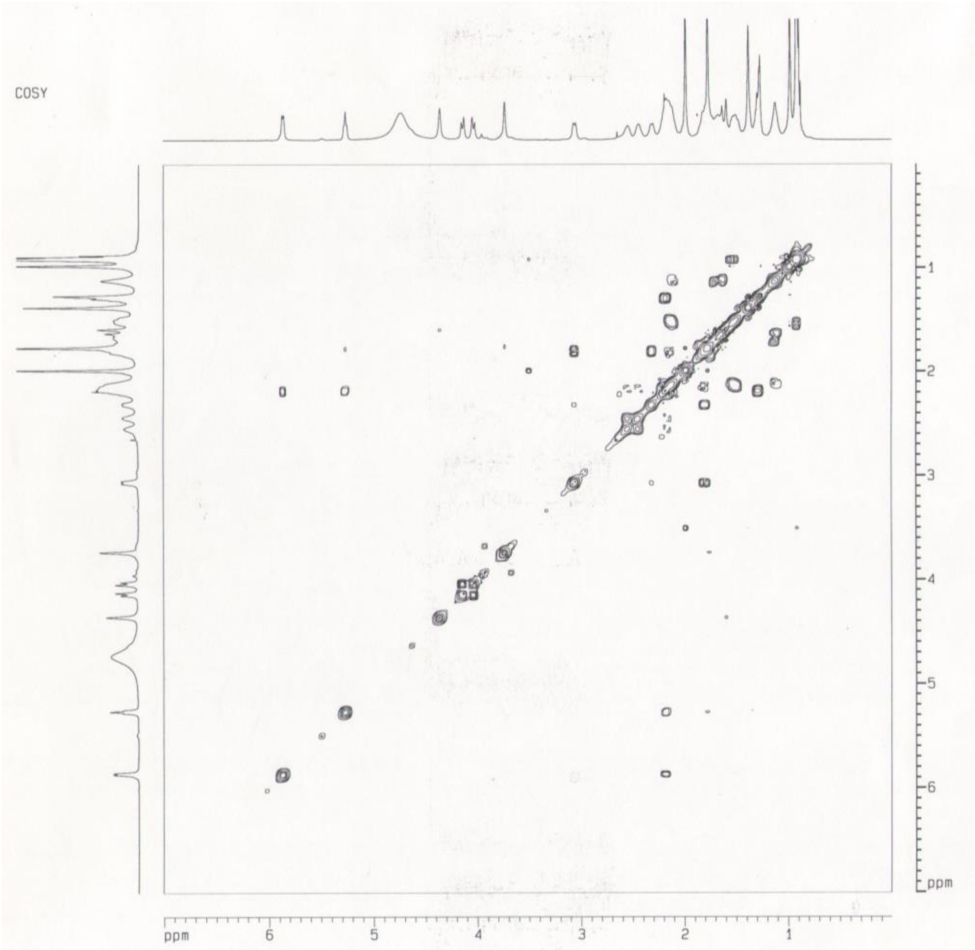

Figure S11. ^1H-^1H COSY spectrum of (24*Z*)- 26-hydroxyfusidic acid (**3**).

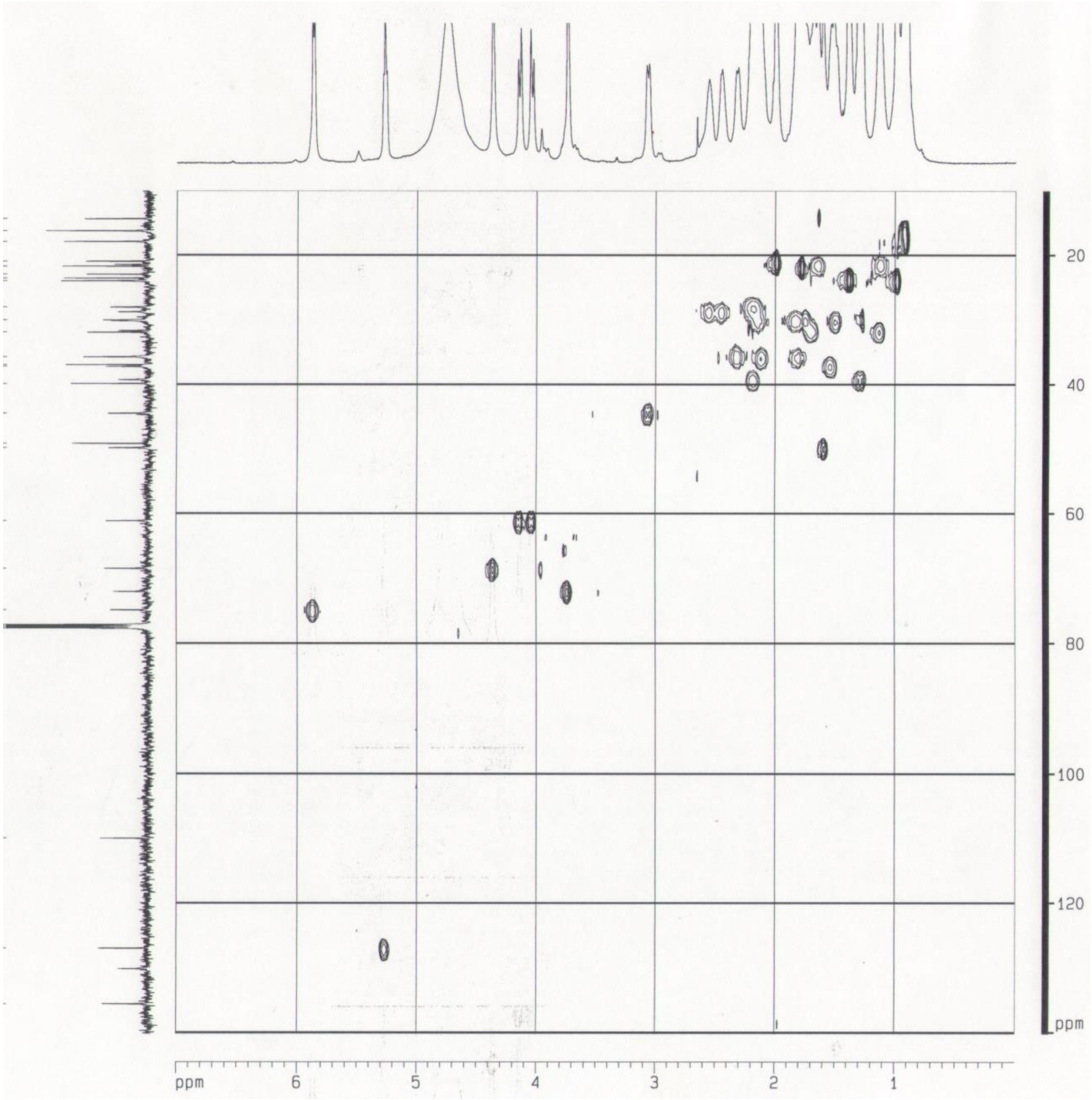

Figure S12. Gradient HMQC spectrum of (24*Z*)- 26-hydroxyfusidic acid (**3**)

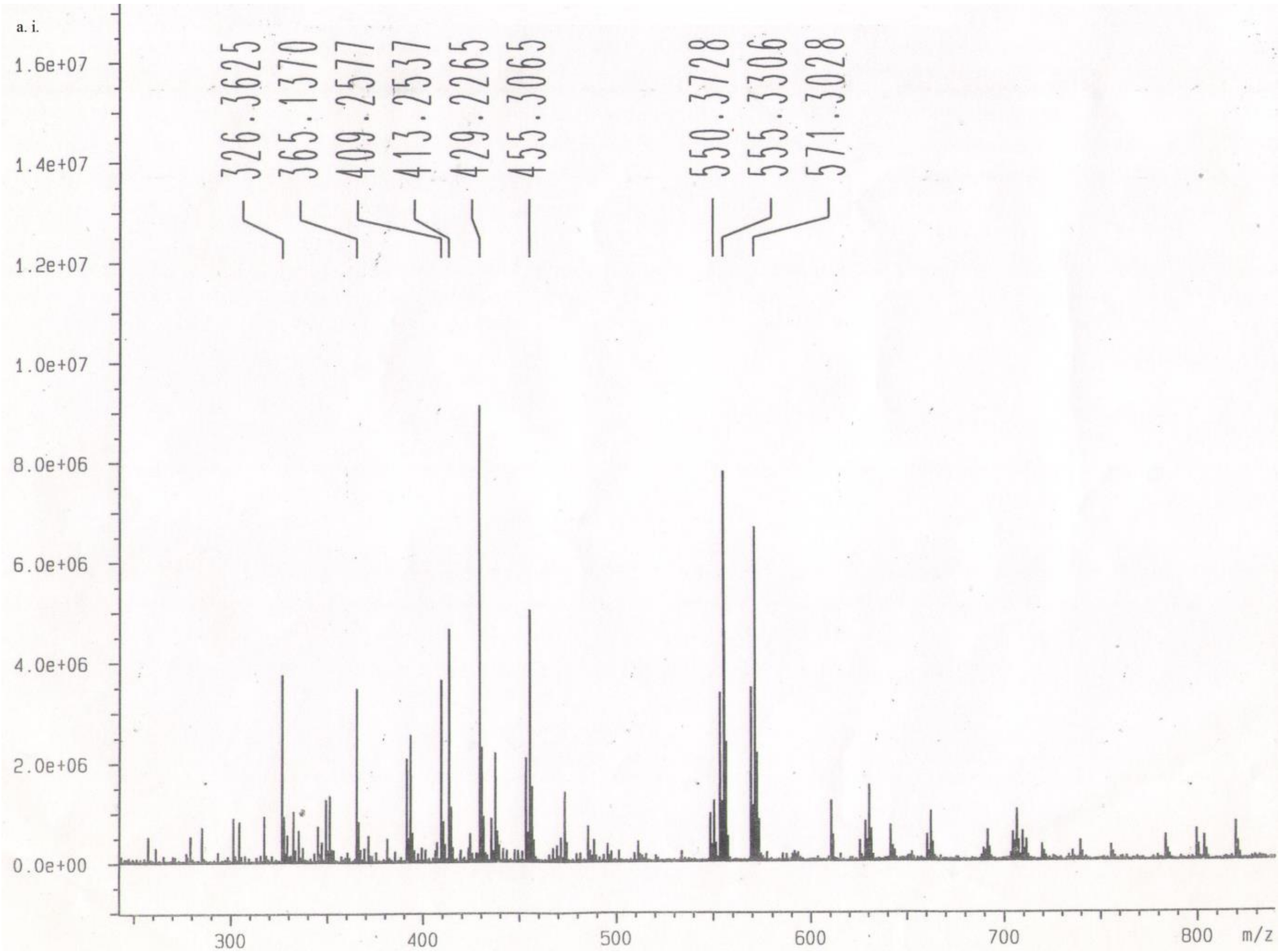

Figure S13. HRESI-MS spectrum of (24*Z*)- 26-hydroxyfusidic acid (**3**)

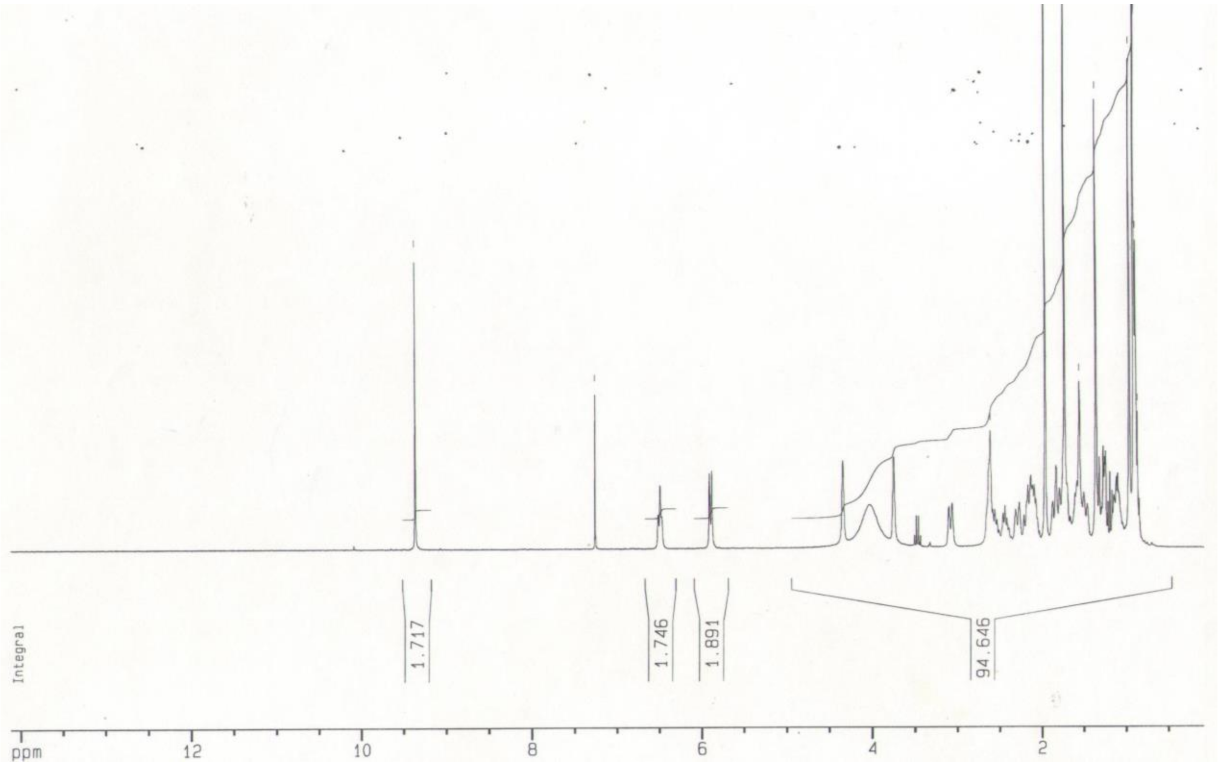

Figure S14. ^{1}H NMR spectrum of 26-formylfusidic acid (**4**), ($CDCl_3$, 300 MHz)

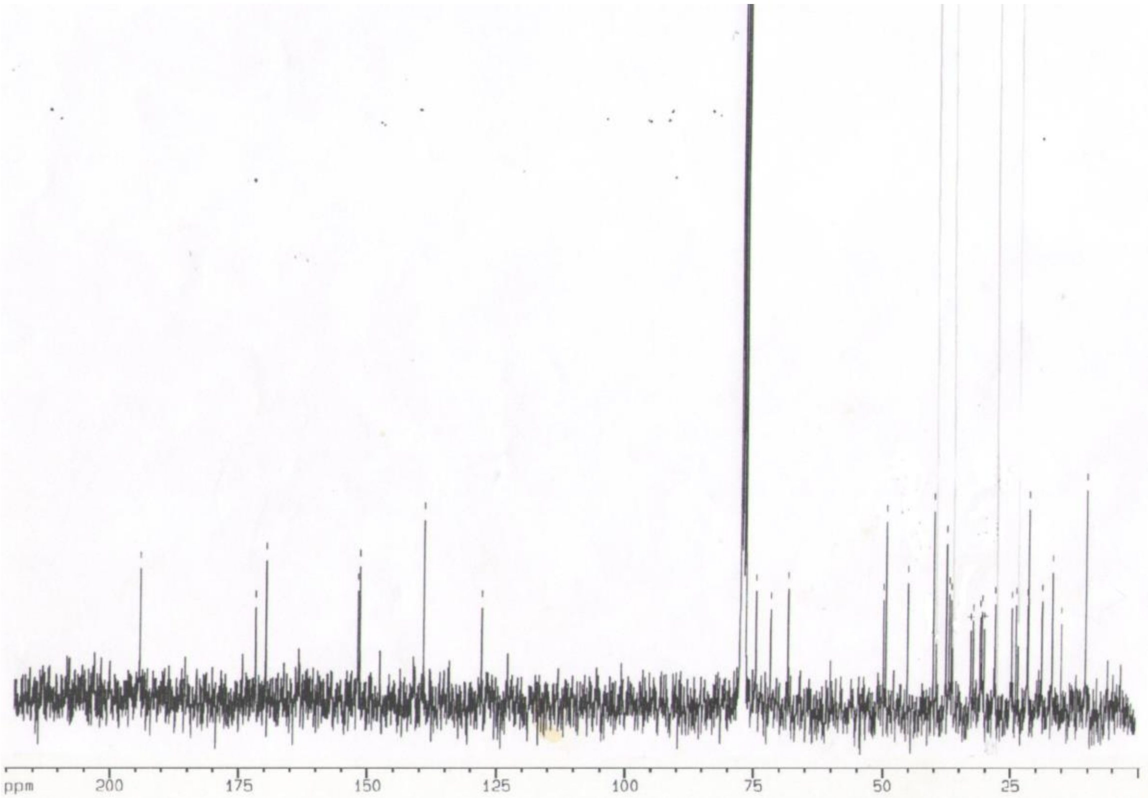

Figure S15. ^{13}C NMR spectrum of 26-formylfusidic acid (**4**), ($CDCl_3$, 75 MHz)

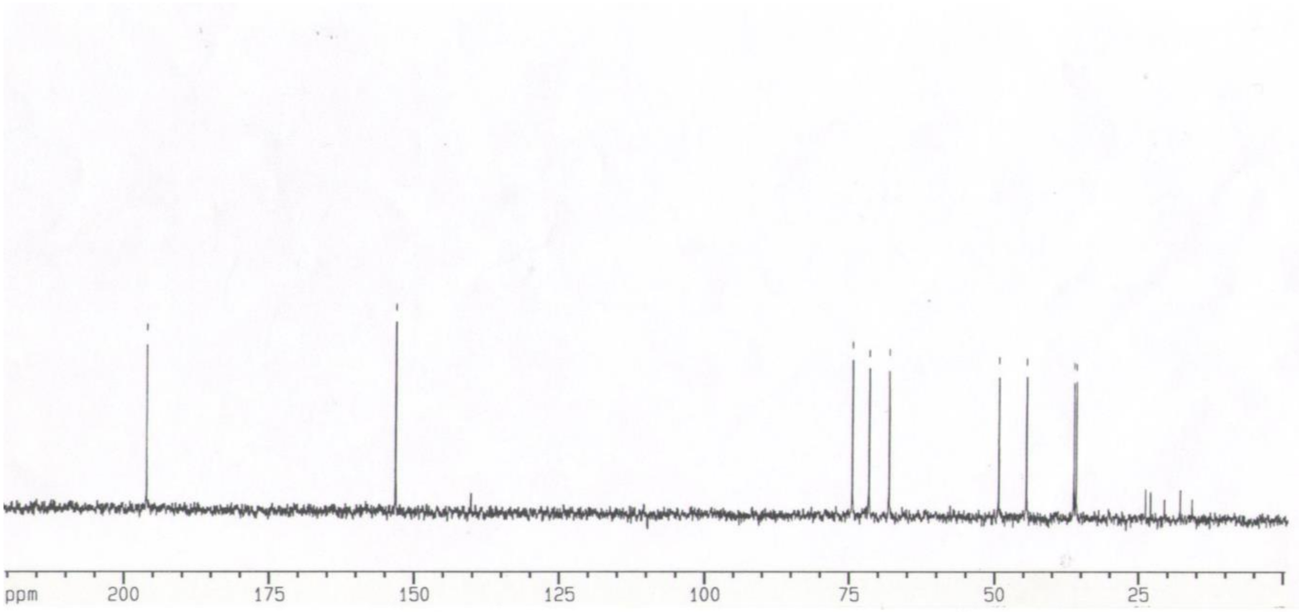

Figure S14. DEPT 90 spectrum of 26-formylfusidic acid (**4**)

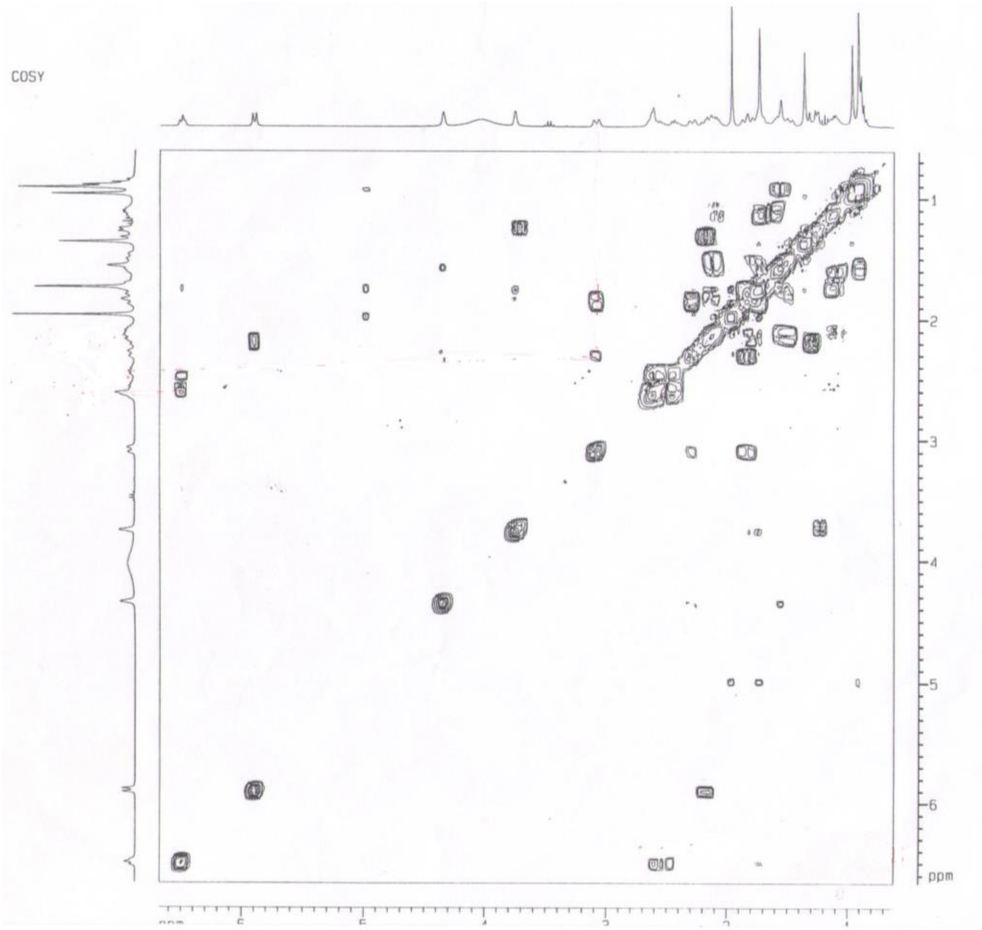

Figure S17. ^{1}H-^{1}H COSY spectrum of 26-formylfusidic acid (**4**)

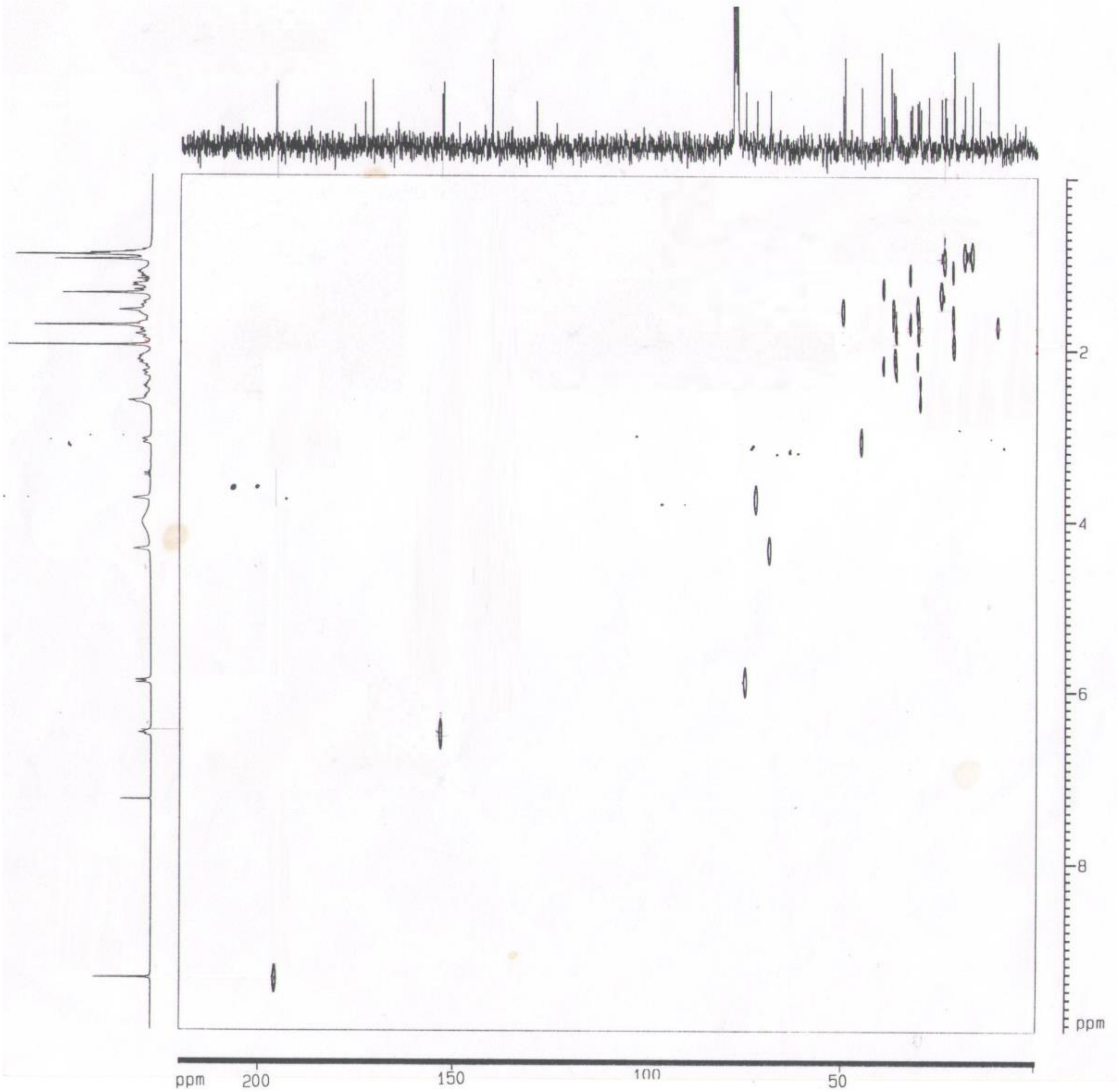

Figure S18. HSQC spectrum of 26-formylfusidic acid (**4**)

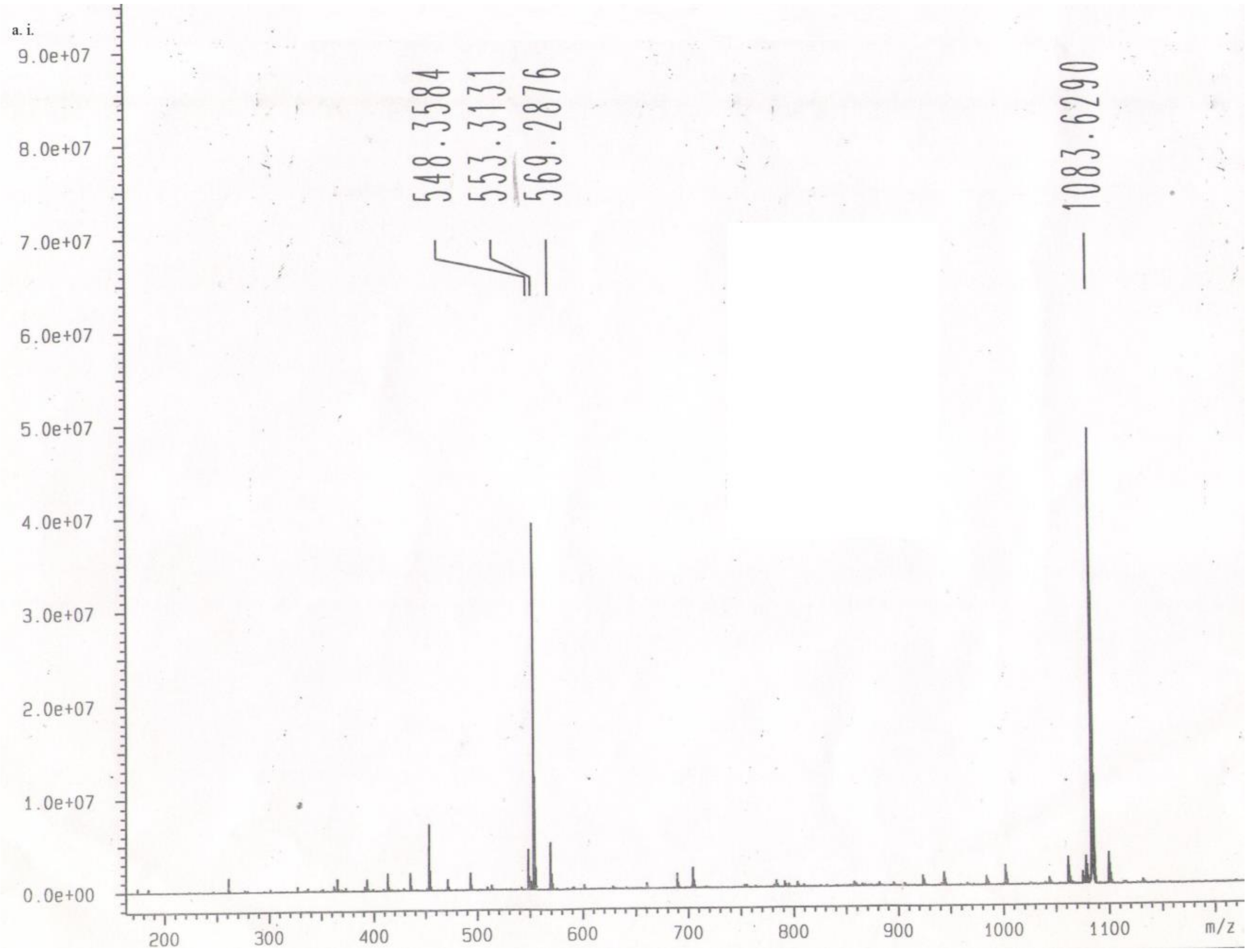

Figure S19. HRESI-MS spectrum of 26-formylfusidic acid (**4**)

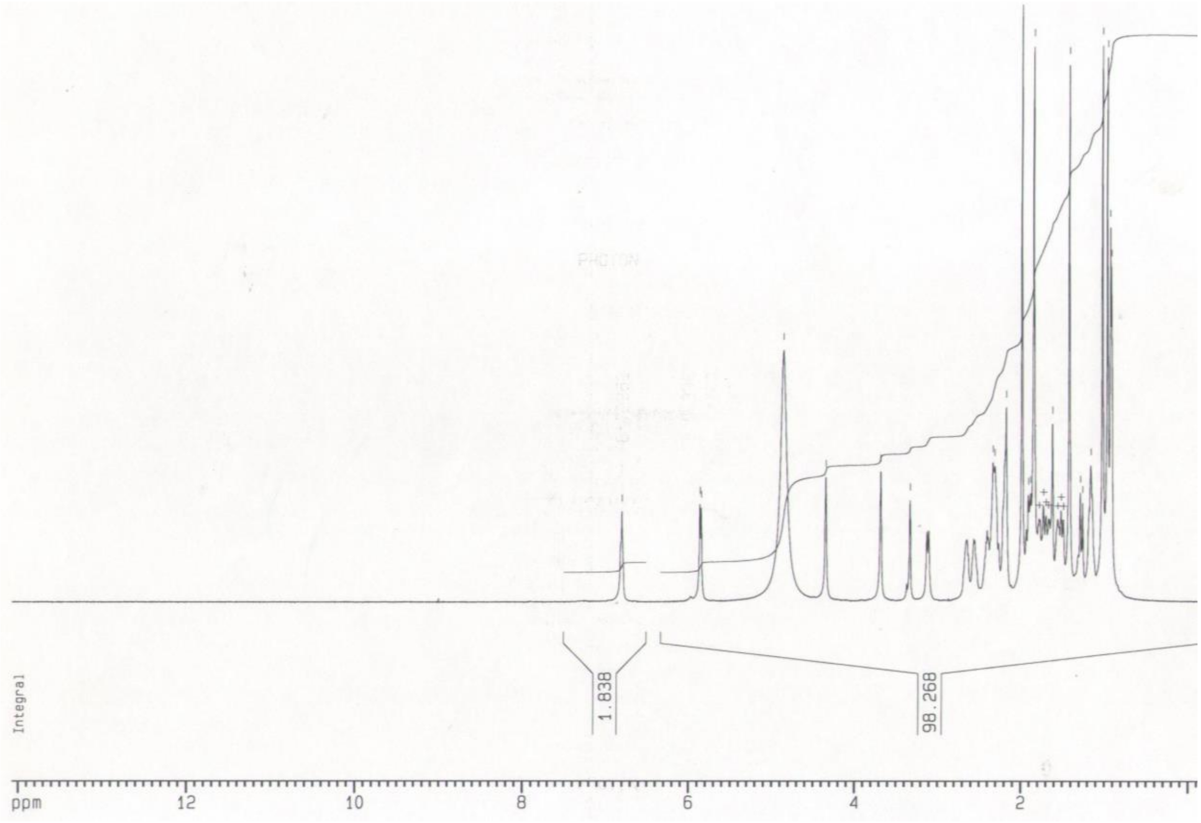

Figure S20. ^{1}H NMR spectrum of 26-carboxyfusidic acid (**5**), (methanol-d_4, 500 MHz)

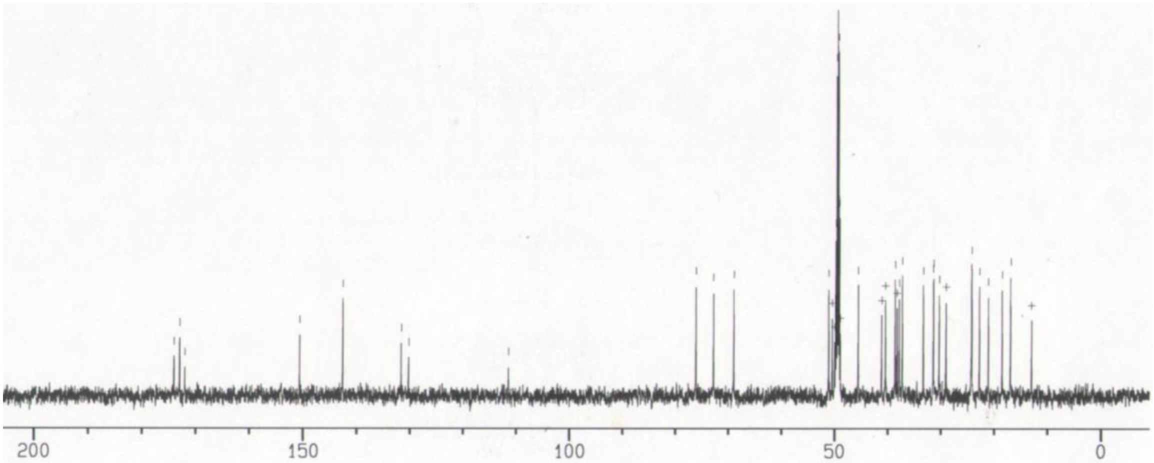

Figure S21. ^{13}C NMR spectrum of 26-carboxyfusidic acid (**5**), (methanol-d_4, 125 MHz)

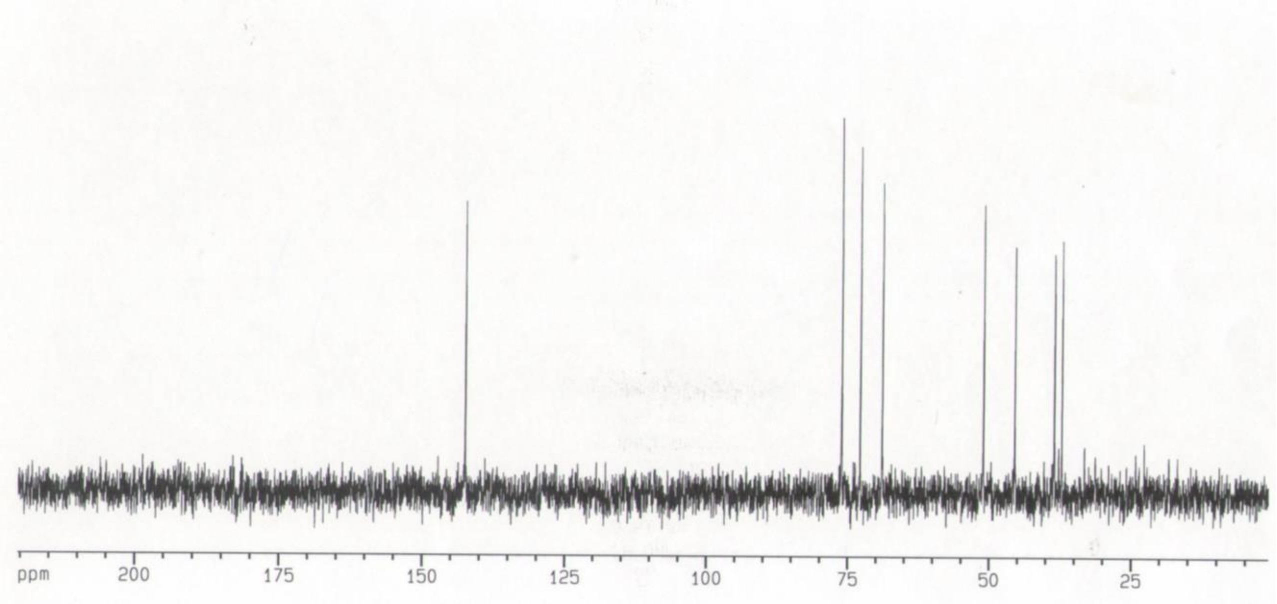

Figure S22. DEPT 90 spectrum of 26-carboxyfusidic acid (**5**)

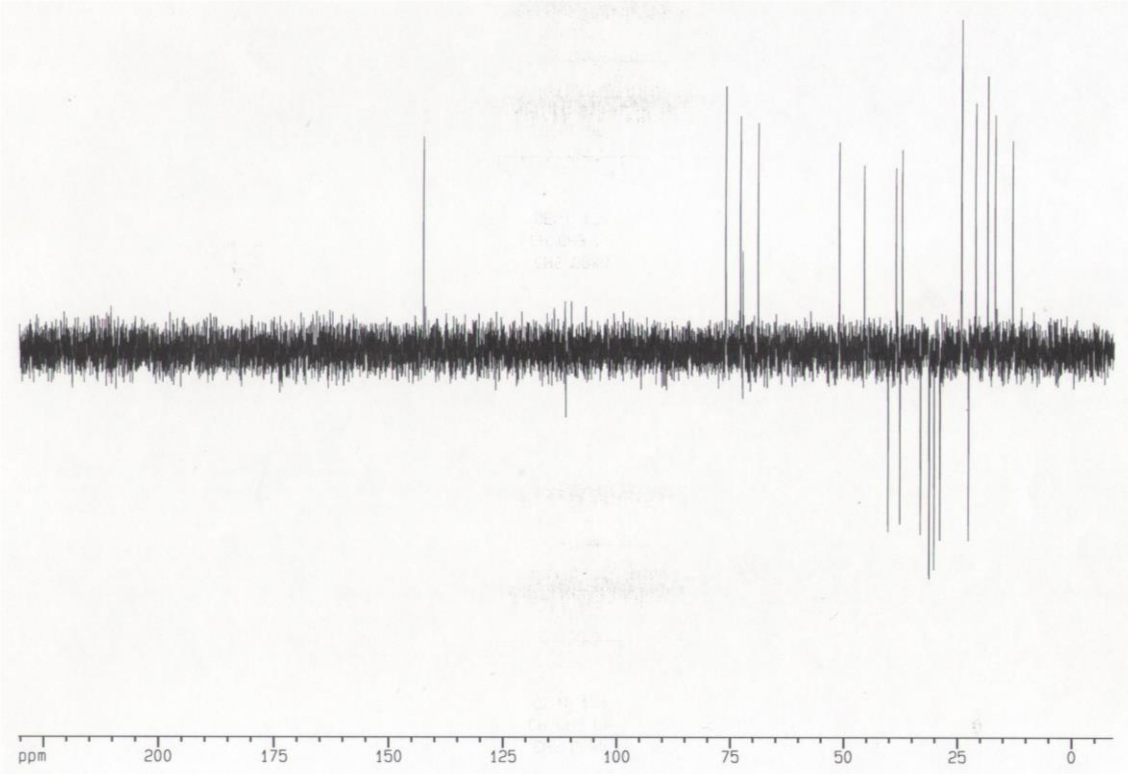

Figure S23. DEPT 135 spectrum of 26-carboxyfusidic acid (**5**).

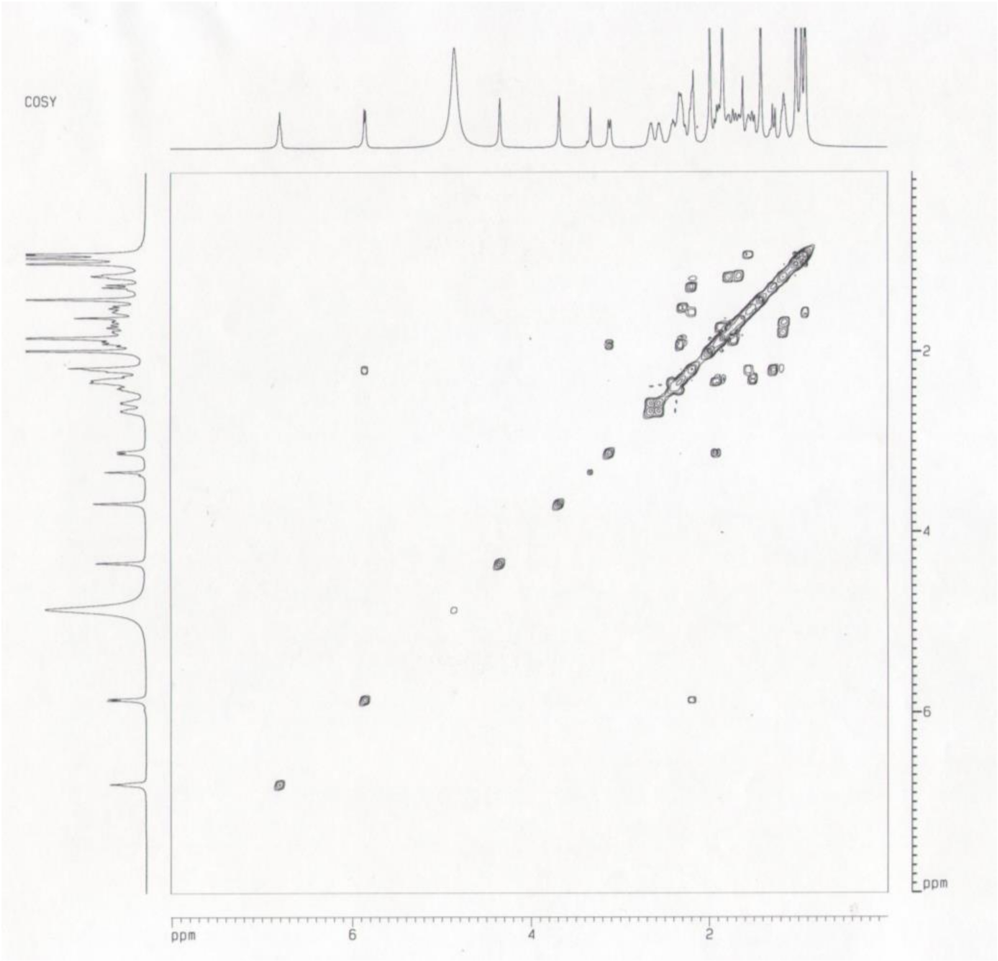

Figure S24. ^{1}H-^{1}H COSY spectrum of 26-carboxyfusidic acid (**5**).

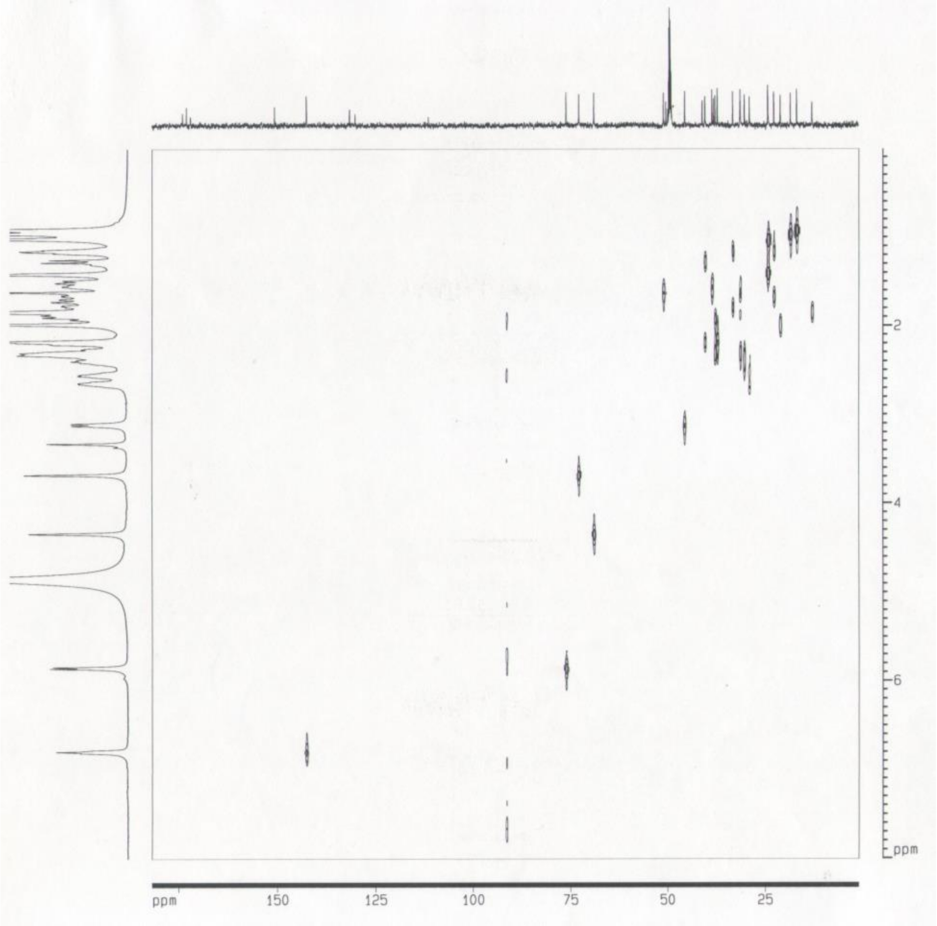

Figure S25. HSQC spectrum of 26-carboxyfusidic acid (**5**)

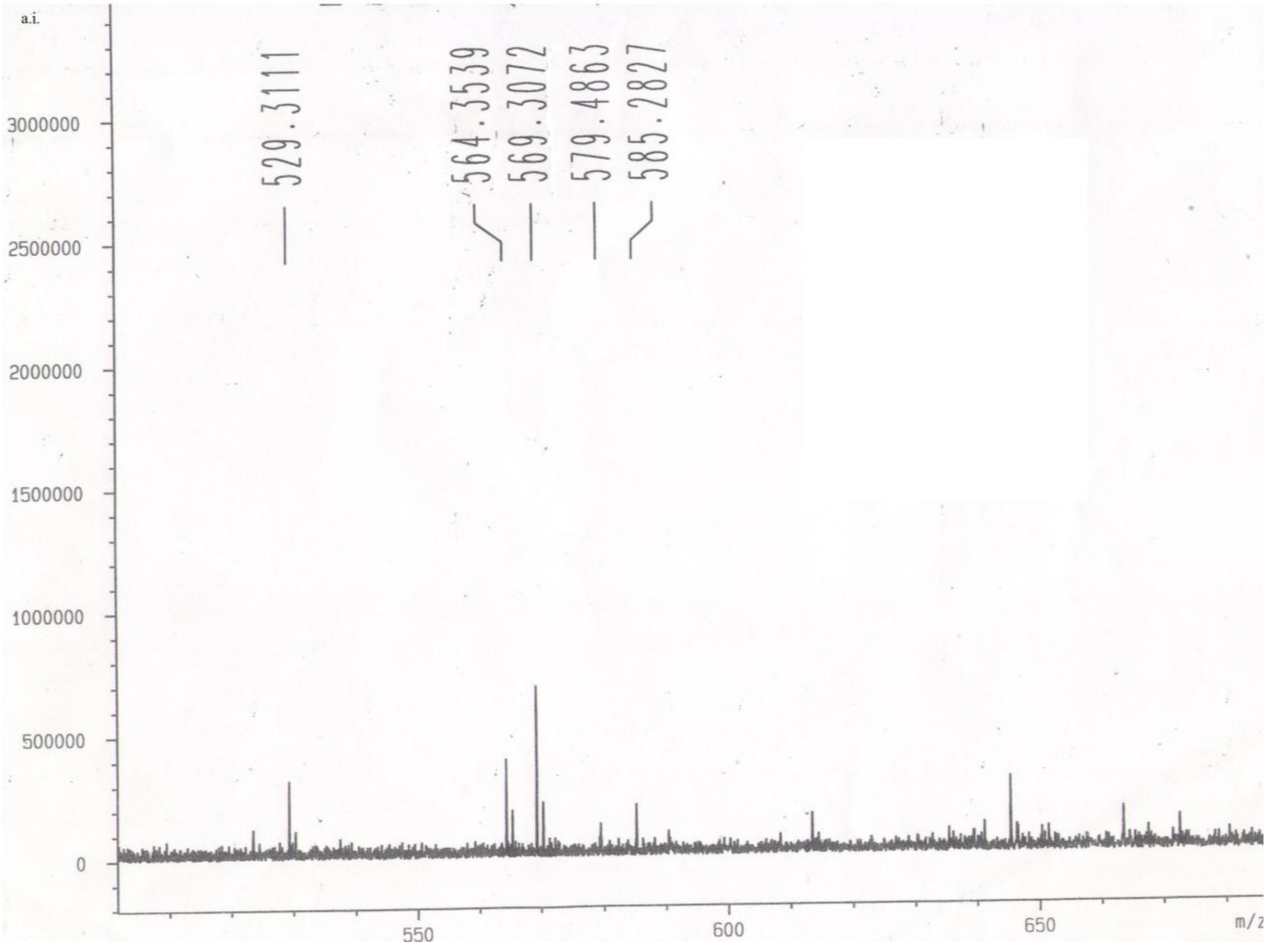

Figure S26. HRESI-MS spectrum of 26-carboxyfusidic acid (**5**)

A list of screened strains:

1- *Aspergillus alliaceous* NRRL 315
2- *Aspergillus flavipes* ATCC 11013
3- *Aspergillus niger* NRRL 599
4- *Aspergillus niger* NRRL 2295
5- *Aspergillus niger* ATCC 10549
6- *Aspergillus ochraceous* NRRL 398
7- *Aspergillus ochraceous* NRRL 405
8- *Candida albicans* (lab isolate)
9- *Cunninghamella bainari* UI 3605
10- *Cunninghamella blackesleeana* 8688a
11- *Cunninghamella blackesleeana* MR 398
12- *Cunninghamella echinulata* NRRL 1382
13- *Cunninghamella elegans* NRRL 1392
14- *Gymnascella citrina* NRRL 6050
15- *Lindera pinnespora* NRRL 2237
16- *Penicillium chrysogenum* ATCC 10002
17- *Penicillium chrysogenum* ATCC 10002-K
18- *Penicillium chrysogenum* ATCC 9480
19- *Penicillium purpureus* UI 193
20- *Penicillium vermiculatum* NRRL 1009
21- *Rhizopus nigricans* NRRL 1477
22- *Rhizopus species* 36060
23- *Rhodotorula rubra* NRRL 1592
24- *Saccharomyces cerivisae* (Baker's yeast)
25- *Streptomyces fulvissimus* NRRL 1453B
